# Deficiency of cannabinoid receptors enhances host susceptibility to bacterial infection

**DOI:** 10.1128/mbio.02088-25

**Published:** 2025-09-18

**Authors:** Hailey A. Barker, Saloni Bhimani, Deyaneira Tirado, Jorge J. Canas, Leandro Nascimento Lemos, Luiz F. W. Roesch, Mariola J. Ferraro

**Affiliations:** 1 Department of Microbiology and Cell Science, Institute of Food and Agricultural Sciences, University of Florida 53701, Gainesville, Florida, USA; 2 Brazilian Center for Research in Energy and Materials (CNPEM) 215006, Campinas, São Paulo, Brazil; University of Wisconsin-Madison, Madison, Wisconsin, USA

**Keywords:** cannabinoid receptors, endocannabinoid system, macrophage, *Salmonella *infection, immune response, microbiome

## Abstract

Host resilience to bacterial infection depends on tightly regulated immune responses, which can be shaped by metabolic cues, including the contribution from bioactive lipids. The endocannabinoid system (ECS), a lipid signaling network known for its neuromodulatory roles, also influences immunity; however, the receptor-specific contributions of cannabinoid receptor 1 (CB1R) and cannabinoid receptor 2 (CB2R) in host–pathogen interactions remain incompletely defined in this context. Using receptor-deficient mouse models, we investigated how CB1R and CB2R modify immune responses to *Salmonella* Typhimurium. CB1R-deficient (CB1R-KO) mice exhibited heightened systemic inflammation, impaired bacterial clearance, and reduced survival in systemic infection, associated with dysregulated macrophage polarization and diminished neutrophil recruitment. In contrast, CB2R-KO mice showed increased susceptibility in both systemic and mucosal infection models, marked by a pro-inflammatory macrophage profile, enhanced neutrophilia, and microbiota dysbiosis. Shotgun metagenomic analysis revealed a reduced abundance of specific protective commensals and altered microbial metabolic pathway profiles in CB2R-KO mice, suggesting a role for CB2R in maintaining mucosal immune–microbiota homeostasis. Collectively, these findings highlight non-redundant roles for CB1R and CB2R in regulating immune dynamics and salmonellosis disease severity, and they point to the ECS as a potential target for host-directed immunomodulatory therapies.

**IMPORTANCE:**

Effective immunity against bacterial pathogens requires a delicate balance between microbial clearance and the containment of inflammatory damage encountered during many infections. The molecular pathways that regulate this equilibrium remain incompletely defined, and the involvement of bioactive lipid signaling mechanisms also needs to be better described. Here, we show that the endocannabinoid receptors CB1R and CB2R play non-redundant roles in host defense against *Salmonella* infection. CB1R deficiency results in exacerbated systemic inflammation, defective bacterial clearance, and dysregulated macrophage polarization. In contrast, CB2R deficiency leads post-infection to gut dysbiosis and has been found to negatively affect the outcome for the host in both systemic and mucosal infection with *Salmonella*. By describing cannabinoid receptor-specific contributions to immune regulation and microbiota dynamics, our findings reveal a previously underappreciated axis of host–pathogen interaction. This study broadens our understanding of lipid-mediated immune modulation and identifies CB1R and CB2R as potential targets for therapies aimed at restoring immune homeostasis and improving infectious disease outcomes.

## INTRODUCTION

The endocannabinoid system (ECS) has a significant role in regulating immune homeostasis, inflammation, and cellular metabolism (1). The ECS comprises the cannabinoid receptors CB1R and CB2R, their endogenous ligands anandamide (AEA) (2) and 2-arachidonoylglycerol (2-AG) (3, 4), and enzymatic pathways controlling their synthesis and degradation (1). While CB1R is predominantly expressed in the central nervous system (CNS), growing evidence supports its immunomodulatory roles in peripheral tissue immune and other cells (5–7). CB2R, by contrast, is highly expressed in immune tissues and is a well-established regulator of macrophage activation, cytokine production, and pathogen control (6–13). Beyond endogenous ligands, specific synthetic plant-sourced cannabinoids—such as Δ9-tetrahydrocannabinol (THC), a partial agonist of both CB1R and CB2R—can also engage the ECS (2). Clinical use of medical cannabis has been associated with improved quality of life in individuals with chronic inflammatory and autoimmune conditions (14). Therefore, there is an expectation that the use of these products might increase with time for both medical and recreational purposes (15, 16). However, unfortunately, a lot of details are lacking in terms of how the stimulation of the ECS might affect host susceptibility or resolution of infection (17). Mechanistic studies indicate that CB2R activation promotes anti-inflammatory, M2-like macrophage polarization and tissue repair, while CB1R functions in systemic immune responses remain incompletely defined (10, 12–14, 18, 19). Understanding how CB1R and CB2R individually contribute to immune control is particularly important in bacterial infections, where successful host defense requires tight regulation of inflammation and microbial clearance. Interestingly, cannabinoid signaling during infection has yielded inconsistent outcomes across pathogens and models, with reports of both enhanced resistance (20) and impaired immunity (20–31) to the bacterial pathogens. These discrepancies emphasize the need to define receptor-specific effects in different bacterial infections, as recently highlighted in a review of ECS–pathogen interactions (32).

*Salmonella enterica*, a Gram-negative facultative intracellular pathogen, presents a significant challenge to host immunity due to its ability to evade and manipulate antimicrobial defenses (19–27). Among the host pathways it targets, lipid signaling has emerged as one of the regulators of immune cell activation and host–pathogen interactions (28–33). Bioactive lipids, including eicosanoids and specialized pro-resolving mediators, actively modulate inflammation and its resolution (34). *Salmonella* can subvert host lipid metabolism—particularly eicosanoid pathways—to promote intracellular survival and suppress immune activation (28, 35). The ECS represents a parallel lipid-derived signaling network with comparable immunomodulatory potential (17). Our previous work demonstrated that *Salmonella* infection perturbs host lipid metabolism, including changes in eicosanoid production that overlap with endocannabinoid pathways (33–35). Markedly, we found that *Salmonella*-infected macrophages exhibit reduced activity of α/β-hydrolase domain-containing 6 (ABHD6) and fatty acid amide hydrolase (FAAH), two key enzymes responsible for degrading the endocannabinoid 2-arachidonoylglycerol (2-AG) (17). These changes suggest that infection elevates intracellular 2-AG levels, which may influence innate immune responses. While 2-AG has been shown to enhance the phagocytosis of zymosan particles, its role in the context of Gram-negative bacterial infection remains undefined (17). Additionally supporting a role for endocannabinoid signaling in antimicrobial defense, elevated levels of 2-AG in a murine model were shown to protect against gastrointestinal infection by *Citrobacter rodentium*, suggesting that ECS modulation may even counteract bacterial virulence mechanisms (32). Despite these insights, the receptor-specific contributions of CB1R and CB2R in immune regulation during *Salmonella* infection, particularly with respect to immune dynamics, remain unclear. Due to the established importance of lipid signaling in immunity and infection, dissecting the roles of CB1R and CB2R in the host response to bacterial pathogens is highly relevant to the fields of immunology, microbiology, and host–pathogen interactions.

In this study, we used CB1R- and CB2R-deficient mouse models to define non-redundant roles of each receptor during *Salmonella* infection. We used both systemic and colitis-associated models (36) to capture distinct inflammatory environments and disease trajectories affected by these receptors (36). Through a set of *in vivo* and *in vitro* experimental approaches, we found that CB1R limits systemic inflammation and supports bacterial clearance, while CB2R consistently preserves mucosal immune homeostasis and affects gut microbiota composition after infection. These findings provide mechanistic understanding into ECS-driven host–pathogen interactions and support CB1R and CB2R as new potential immunological checkpoints during enteric infection.

## RESULTS

### Gene expression analysis of mouse endocannabinoid receptors (*cnr1* and *cnr2*) in *Salmonella*-infected macrophages

Prior studies have shown that endocannabinoid hydrolases, such as fatty acid amide hydrolase (FAAH) and α/β-hydrolase domain-containing 6 (ABHD6), are downregulated in macrophages infected with *Salmonella*, suggesting an infection-induced elevation of intracellular endocannabinoid levels (27). To determine whether this regulatory effect extends to cannabinoid receptors, we analyzed the expression of *cnr1* (CB1R protein) and *cnr2* (CB2R protein) in bone marrow-derived macrophages (BMDMs) infected with *Salmonella enterica* serovar Typhimurium. Both *cnr1* and *cnr2* transcripts were significantly downregulated at 2 and 24 hours post-infection (hpi) (Fig. S1A and B). To test whether this pattern extends to human macrophages and other *Salmonella* serovars, we analyzed publicly available RNA-seq data (37), in which transcriptomes were profiled from distinct THP-1 macrophage populations infected with *Salmonella* Typhi for 18 hours. These populations were stratified based on bacterial viability and replication status (naïve, bystander, host-killed, non-replicating, and replicating). Notably, *cnr1* expression was significantly elevated in macrophages containing actively replicating *S.* Typhi compared to those with host-killed bacteria (~2-fold, *P* < 0.05). However, *cnr1* was downregulated in THP-1 cells challenged with *S.* Typhi compared to uninfected cells, as well as in macrophages that had killed *S.* Typhi compared to naïve cells (Fig. S1C). In contrast, *cnr2* expression showed minimal variation across these conditions in these human macrophages infected with *S*. Typhi, with no significant induction. Overall, these findings indicate that endocannabinoid receptor expression is regulated in response to *Salmonella* infection. In mouse macrophages, both *cnr1* and *cnr2* are significantly downregulated, suggesting a general suppression of cannabinoid signaling. In contrast, human macrophages exhibit a more selective response, with *cnr1* expression decreasing specifically in the context of intracellular bacterial replication, while *cnr2* remains largely unchanged. Importantly, the expression of *cnr1* and *cnr2* has not yet been assessed using *in vivo* infection models, and studies are needed to determine if similar regulatory patterns occur in infected animals.

### Cannabinoid receptor deficiency leads to differential recruitment of lymphoid and myeloid immune cells during murine *Salmonella* challenge

**Fig 1:**
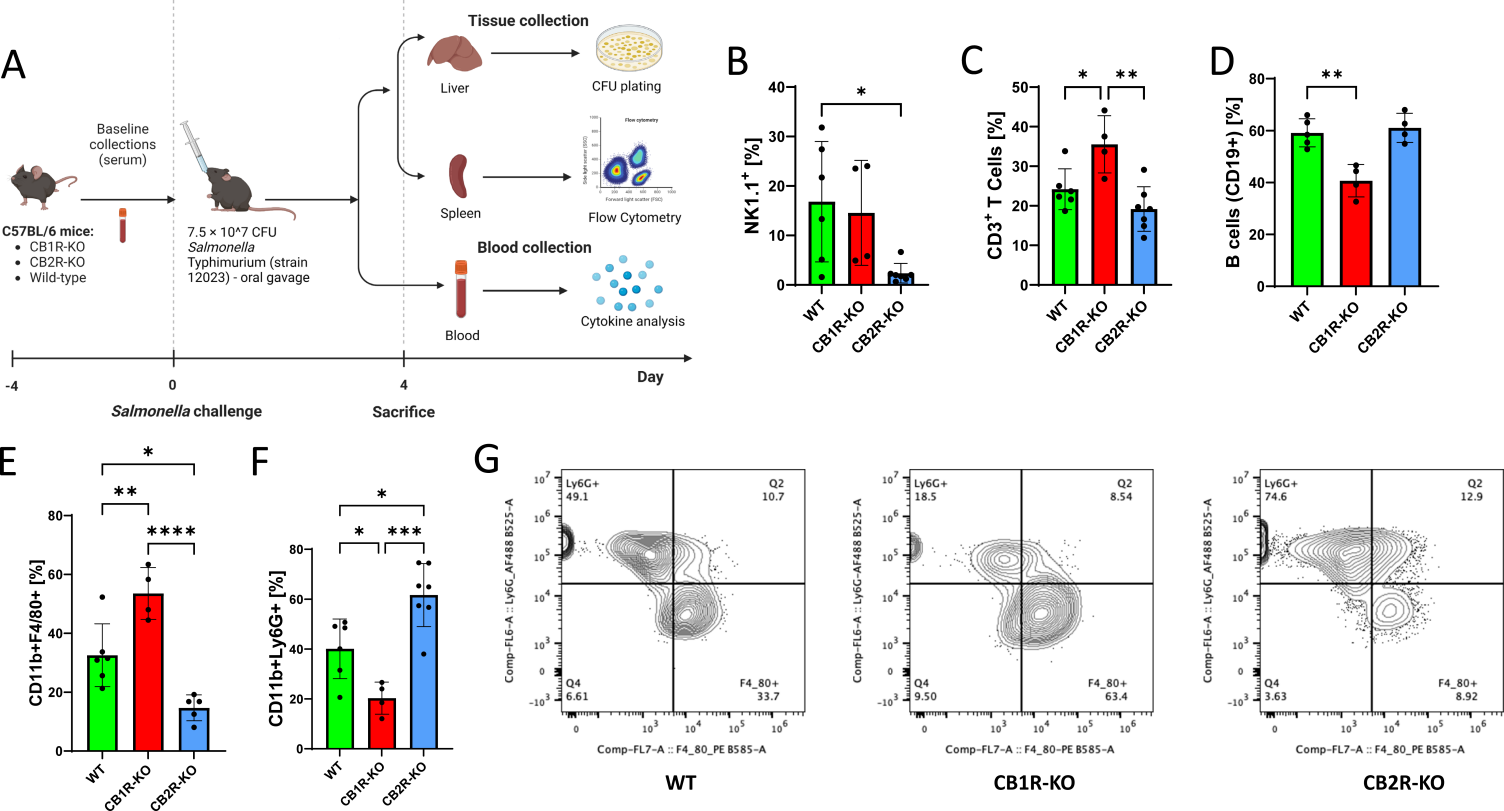
Roles of CB1R and CB2R deficiencies in modulating host resilience and survival against *Salmonella* Typhimurium infection in C57BL/6 mice. (**A**) Experimental setup illustrating the oral infection of wild-type (WT), CB1 receptor knockout (CB1R-KO), and CB2 receptor knockout (CB2R-KO) C57BL/6 mice with 7.5 × 10^7^ CFU of *Salmonella* Typhimurium. (**B–F**) Flow cytometry analysis of various immune cell subsets in the spleens of these mice post-infection, showing variations in the innate immune response across the three genotypes. Quantified cell populations include NK cells (**B**), CD3+ T cells (**C**), CD19+ B cells (**D**), macrophages (**E**), and neutrophils (**F**). (**G**) Comparison of macrophage and neutrophil populations across WT, CB1R-KO, and CB2R-KO genotypes. N values are indicated on figures as individual dots representing different experimental animals. One-way ANOVA was used for statistical analysis. Statistical significance is denoted by asterisks: *P < 0.05, **P < 0.01, ***P < 0.001, and ****P < 0.0001.

To investigate how CB1R and CB2R modulate immune cell recruitment *in vivo*, CB1R knockout (CB1R-KO), CB2R knockout (CB2R-KO), and wild-type (WT) C57BL/6 mice were orally infected with *Salmonella* Typhimurium (7.5 × 10⁷ CFU/mouse). Immune responses were assessed at 4 days post-infection (dpi) (Fig. 1A). To minimize microbiota-driven confounding, mice were cohoused prior to infection. Baseline assessments confirmed no differences in body weight (Fig. S2A) or in population of cells like macrophages, B cells, and T cells across genotypes (Fig. S2B). Flow cytometry analysis of splenocytes revealed receptor-specific alterations in both lymphoid and myeloid compartments after *Salmonella* infection. Among lymphoid populations, CB2R-KO mice showed a significant reduction in the percentage of NK cells compared to WT controls (mean decrease of ~60%, *P* < 0.05; Fig. 1B), while CD3^+^ T cells and CD19^+^ B cells were not significantly altered (Fig. 1C and D). In contrast, CB1R-KO mice exhibited a ~32% increase in the frequency of T cells and ~45% decrease in B cell percentages relative to WT controls (Fig. 1C and D). Myeloid cell analysis also revealed genotype-specific effects (Fig. 1E through G). CB1R-KO mice showed a significant 64.5% increase in CD11b^+^F4/80^+^ macrophage frequency, whereas CB2R-KO mice showed a 54.9% reduction relative to WT (Fig. 1E and G). Conversely, CD11b^+^Ly6G^+^ neutrophil frequencies were reduced by 49.3% in CB1R-KO mice and elevated by 53.8% in CB2R-KO mice (Fig. 1F), indicating different patterns of myeloid cell recruitment depending on receptor deficiency after *Salmonella* infection. These results indicate receptor-specific alterations in myeloid cell recruitment, with CB1R deficiency associated with increased macrophage and decreased neutrophil accumulation, and CB2R deficiency showing the opposite pattern. To determine whether these differences were infection specific, we examined splenic immune populations in uninfected mice. No significant genotype-dependent differences in myeloid or lymphoid cell frequencies were observed at baseline (Fig. S2B), suggesting that the immune alterations are induced under bacterial stimuli. These trends were recapitulated in the streptomycin-pretreated colitis model. At 3 dpi, CB2R-KO mice again exhibited decreased splenic macrophage frequencies and increased neutrophils (Fig. S3A through C) in the colitis model of salmonellosis. CB1R-KO mice in this model showed only modest reductions in macrophages, with no significant changes in neutrophils.

Together, these findings demonstrate that CB2R plays a consistent role in regulating macrophage and neutrophil responses during both systemic and mucosal *Salmonella* infection. In contrast, CB1R effects appear to be model-dependent, with more pronounced alterations observed in the systemic infection model. However, further studies are needed to track the *in vivo* expression patterns of these receptors across different immune cell types and tissues.

### Cannabinoid receptor 1 and 2 deficiencies shape macrophage polarization states and systemic immune responses in murine models of *Salmonella* infection

**Fig 2:**
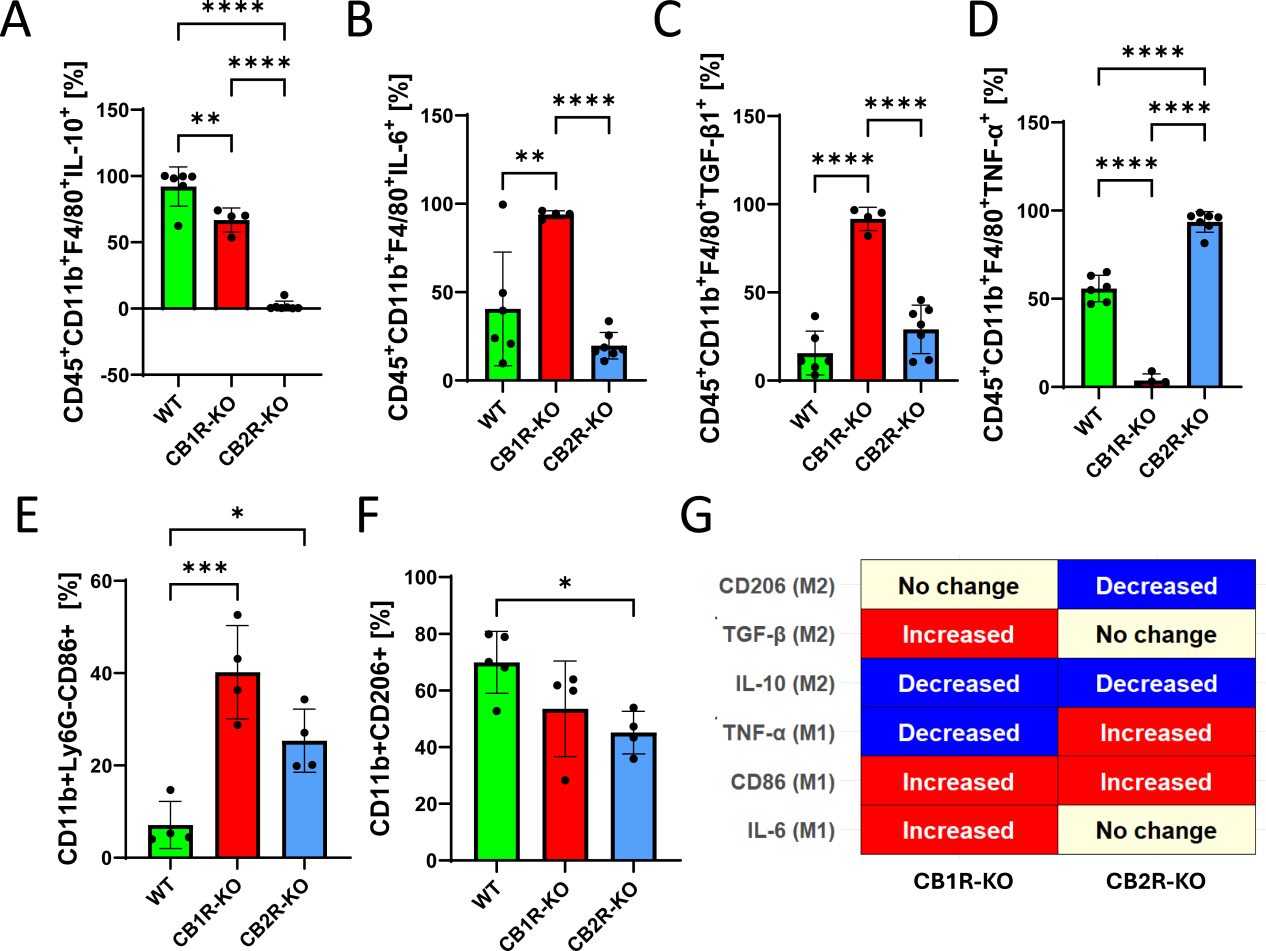
Cytokine expression in splenic macrophages in CB1R and CB2R knockout mice post-infection with *Salmonella* Typhimurium. Flow cytometry was used to quantify cytokine production in splenic macrophages (CD45^+^CD11b^+^F4/80^+^) from wild-type (WT), CB1 receptor knockout (CB1R-KO), and CB2 receptor knockout (CB2R-KO) C57BL/6 mice, 4 days post-infection with *Salmonella* Typhimurium. The following markers were analyzed: (**A**) IL-10, (**B**) IL-6, (**C**) TGF-β, (**D**) TNF-α, (**E**) CD86 and (**F**) CD206. Each bar graph represents the mean percentage ± SEM, with individual data points for each mouse. (**G**) Graphical summary of M1/M2 macrophage polarization markers in CB1R-KO and CB2R-KO splenic macrophages post-*Salmonella* infection, highlighting the receptor-specific shifts in immune phenotypes. N values are indicated on figures as individual dots representing different experimental animals. Statistical analyses were performed using one-way ANOVA to compare cytokine and marker expression levels between WT, CB1R-KO, and CB2R-KO groups. Statistical significance is denoted by asterisks: *P < 0.05, **P < 0.01, ***P < 0.001, and ****P < 0.0001.

Given the observed differences in immune cell recruitment between *Salmonella*-infected CB1R-KO and CB2R-KO mice relative to WT controls, we next assessed cytokine production and macrophage polarization states in the spleens of infected animals at 4 days post-infection (dpi) (Fig. 2). Flow cytometry analysis revealed that splenic macrophages from CB1R-KO mice produced significantly less IL-10 (Fig. 2A), while exhibiting elevated levels of IL-6 (Fig. 2B) and TGF-β (Fig. 2C) compared to WT controls. In CB2R-KO mice, macrophages similarly showed reduced IL-10 and increased TGF-β expression (Fig. 2C); however, IL-6 levels remained comparable to WT (Fig. 2B). Interestingly, intracellular TNF-α levels showed opposing trends in the two knockout genotypes: CB1R-KO macrophages displayed reduced TNF-α, while CB2R-KO macrophages exhibited elevated TNF-α compared to control-infected mice (Fig. 2D). It is important to note that these measurements reflect intracellular cytokine levels and may not directly correlate with cytokine secretion. Surface marker analysis further distinguished their polarization states. CB1R-KO macrophages upregulated CD86 but retained CD206 expression, consistent with a mixed M1/M2b, but primarily M2b phenotype (Fig. 2E and F). In contrast, CB2R-KO macrophages showed elevated CD86 and significantly reduced CD206, aligning with a classical M1 profile (Fig. 2E and F). These phenotypes are summarized in Fig. 2G.

**Fig 3:**
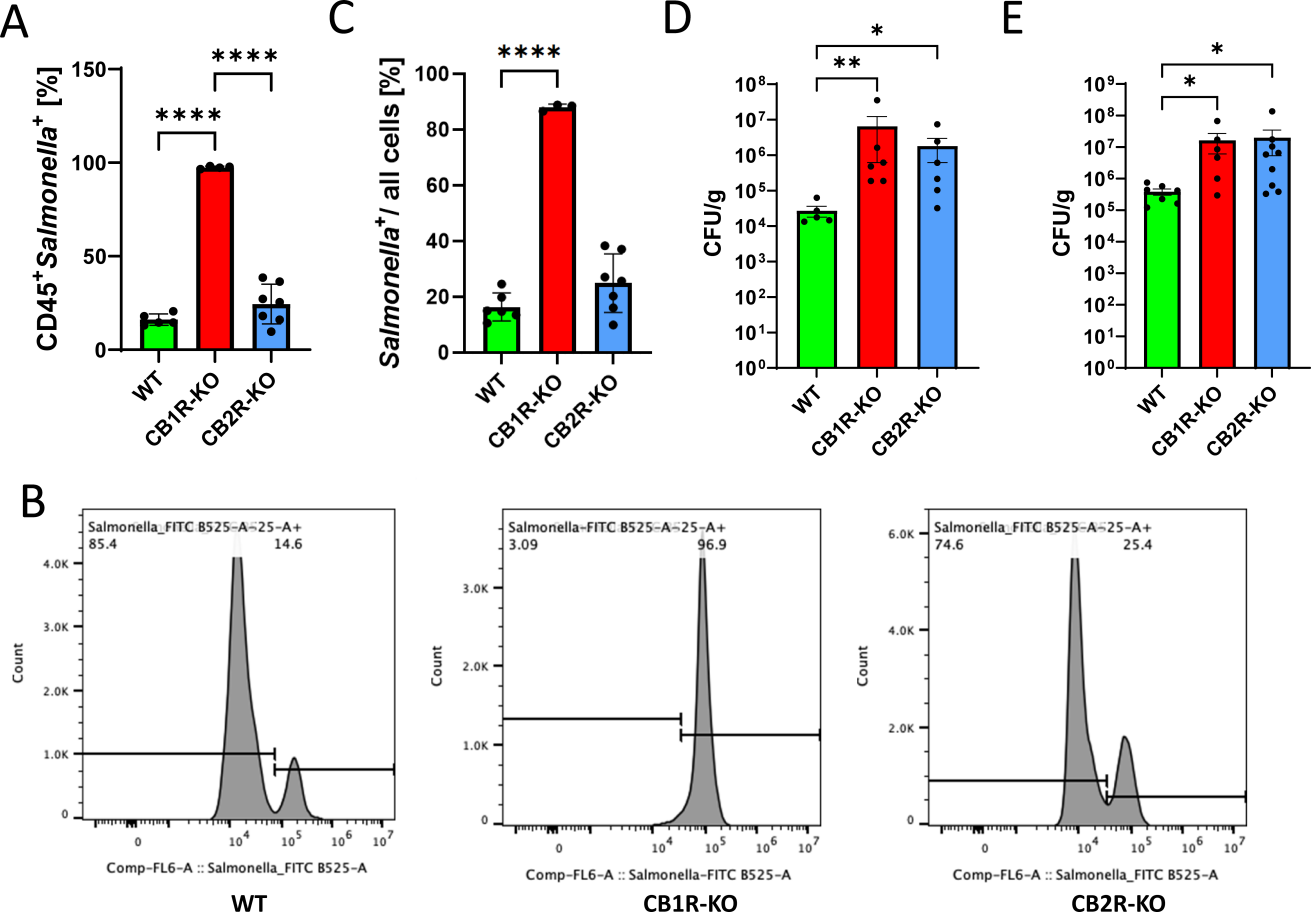
Influence of cannabinoid receptors on *Salmonella* Typhimurium dissemination in C57BL/6 mice. (**A–C**) Flow cytometry analysis of splenic cells from C57BL/6 mice infected with *Salmonella* Typhimurium to assess bacterial dissemination. (**A and B**) Data represent CD45^+^ *Salmonella*^+^ cells, while (**C**) analysis expands to all live cells, mapping the systemic spread of *Salmonella* in CB1R and CB2R knockout (CB1R-KO and CB2R-KO) mice compared to controls. (**D and E**) Colony-forming unit (CFU) plating of the liver (**D**) or spleen (**E**) from infected mice to quantify viable *Salmonella* loads. For panels **A**–**C**, statistical analysis was performed using one-way ANOVA followed by Tukey’s *post hoc* test. Statistical significance is indicated by asterisks: *P < 0.05, ****P < 0.0001. For panels **D** and **E**, CFU data were analyzed using one-way ANOVA assuming a lognormal distribution; variance homogeneity was evaluated using Brown–Forsythe and Bartlett’s tests. N values are indicated as individual dots representing different experimental animals. Statistical significance is indicated by asterisks: *P < 0.05, **P < 0.01, ****P < 0.0001.

To determine whether the observed immune phenotypes were associated with altered control of *Salmonella* infection, we assessed bacterial burden in the spleen using both flow cytometry and CFU plating. Flow cytometric analysis revealed a significant increase in CD45^+^ *Salmonella*^+^ splenocytes in CB1R-KO mice (Fig. 3A and B), along with an elevated bacterial load among all live splenocytes (Fig. 3C), indicating impaired systemic containment in the absence of CB1R. These findings were corroborated by CFU assays, which confirmed increased bacterial loads in both the spleen and liver of CB1R-KO mice (Fig. 3D and E). In contrast, CB2R-KO mice showed increased bacterial burden only in CFU assays, suggesting a more modest defect in pathogen clearance (Fig. 3D and E).

To determine whether these macrophage phenotypes and bacterial dissemination patterns were conserved in a mucosal model, we analyzed mice infected using the streptomycin-pretreated colitis model of salmonellosis. At 3 dpi, both knockout strains again displayed elevated TGF-β expression (*P* < 0.0001; Fig. S4A). However, only CB1R-KO macrophages showed a robust increase in IL-6 (*P* < 0.0001; Fig. S4B), mirroring the splenic response. Flow cytometry of peripheral blood at 2 dpi also revealed higher frequencies of *Salmonella*-positive leukocytes in both CB1R-KO and CB2R-KO mice compared to WT (Fig. S4C), along with increased circulating neutrophils (CD45^+^CD11b^+^Ly6G^+^) (Fig. S4D), indicating enhanced systemic inflammation and bacterial dissemination in these animals.

We also performed *in vitro* experiments using murine bone marrow–derived macrophages (BMDMs) isolated from WT, CB1R-KO, and CB2R-KO mice, which were infected with *Salmonella* for 2 hours. CB2R-KO BMDMs consistently produced increased levels of TNF-α, IL-6, and IFN-γ, as assessed by intracellular flow cytometry (Fig. S5A through C) and ELISA of culture supernatants (Fig. S5D). In contrast, CB1R-KO BMDMs showed a selective reduction in IFN-γ (Fig. S5C), with no significant changes in TNF-α or IL-6 at the time point tested. To determine whether baseline inflammatory priming contributed to the heightened cytokine responses in CB2R-deficient macrophages, we performed qPCR on uninfected BMDMs. Among the genes analyzed (*tlr4*, *tnfa*, *arg1*, and *inos*), only *inos* expression was modestly elevated in CB2R-KO cells relative to WT and CB1R-KO macrophages (Fig. S5E), suggesting limited baseline polarization differences in resting cells that could have contributed to the phenotype.

Together, these findings demonstrate that CB1R and CB2R play distinct roles in regulating macrophage function, cytokine production, and host–pathogen interactions during *Salmonella* infection. CB1R deficiency is associated with elevated IL-6, reduced IL-10 and TNF-α, and a skewing toward an M2b macrophage phenotype *in vivo*, contributing to impaired bacterial clearance and heightened systemic inflammation. In contrast, CB2R deficiency promotes a classical M1 macrophage phenotype—characterized by increased TNF-α production and reduced CD206 expression—both *in vivo* and *in vitro*, highlighting its role in driving pro-inflammatory activation. These effects were seen across both systemic and mucosal infection models.

### CB1R- and CB2R-deficient mice exhibit distinct vulnerability profiles during *Salmonella* challenge

**Fig 4:**
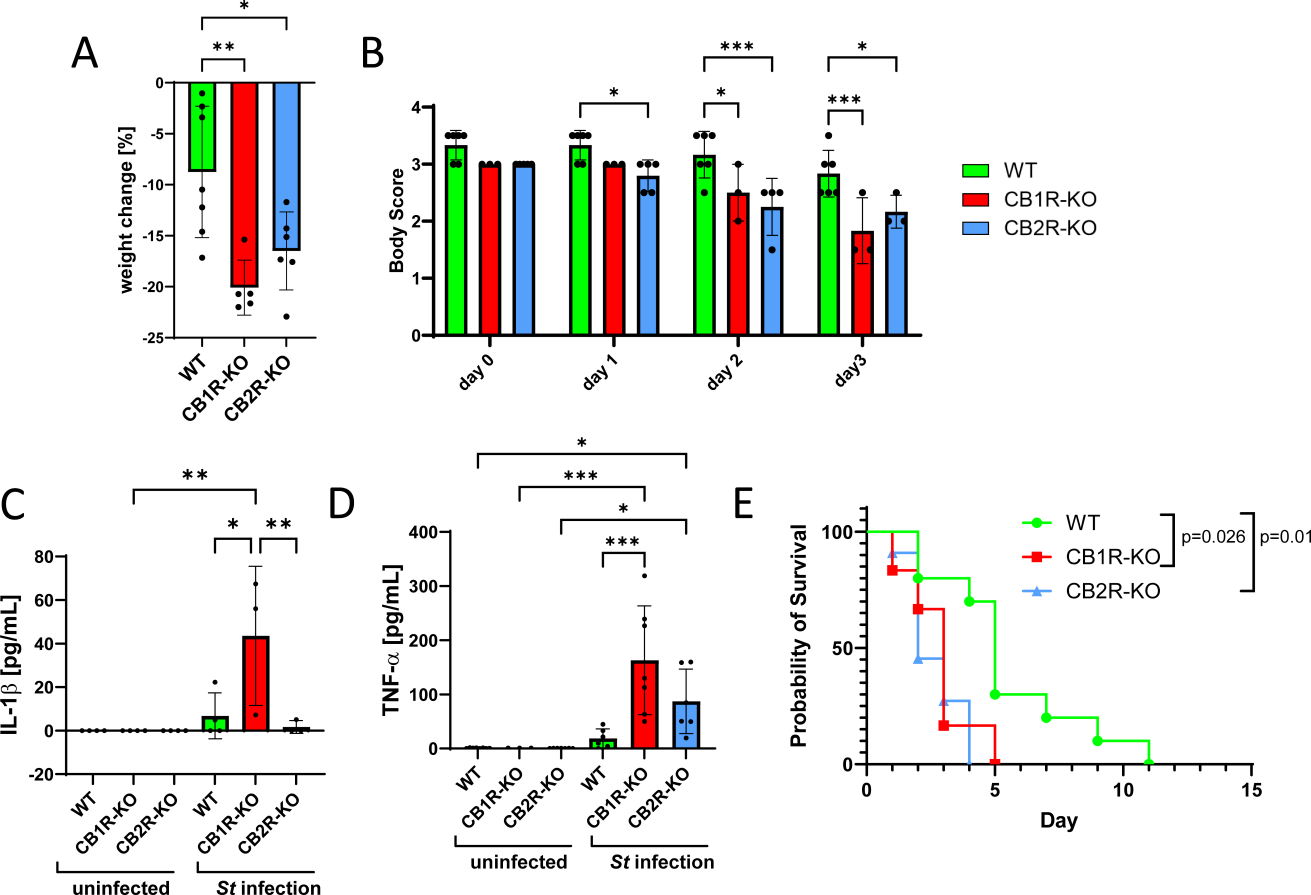
Impact of CB1R and CB2R knockout on *Salmonella* Typhimurium infection outcomes in mice using systemic model of salmonellosis. (**A**) Body weight loss in wild-type (WT), CB1R knockout (CB1R-KO), and CB2R knockout (CB2R-KO) mice at 4 days post-infection (dpi) with *Salmonella* Typhimurium. Data are from two independent cohorts. (**B**) Body condition scores in WT, CB1R-KO, and CB2R-KO mice at 0, 1, 2, and 3 dpi. (**C**) Serum IL-1β levels in WT, CB1R-KO, and CB2R-KO mice at baseline and 4 dpi. Data from one cohort. (**D**) Serum TNF-α levels in WT, CB1R-KO, and CB2R-KO mice at baseline and 4 dpi. Data are from two independent cohorts. (**E**) Kaplan–Meier survival curves for WT (*n* = 10), CB1R-KO (*n* = 6), and CB2R-KO (*n* = 11) mice. Survival differences were analyzed using the log-rank (Mantel–Cox) test. Two independent cohorts were used for WT and CB2R-KO mice. Data are presented as mean ± SEM. N values are indicated on figures as individual dots representing different experimental animals. For (**A**–**D**), one-way ANOVA was used for statistical analysis. Statistical significance is indicated as follows: *P < 0.05, **P < 0.01, and ***P < 0.001.

To evaluate how CB1R and CB2R impact disease severity during systemic *Salmonella* infection, we assessed clinical outcomes in CB1R-KO, CB2R-KO, and WT mice following oral challenge with *S.* Typhimurium (7.5 × 10⁷ CFU). Parameters included weight loss, body condition, serum cytokines, and survival. Compared to WT mice, CB1R-KO and CB2R-KO animals experienced significantly greater weight loss (*P* = 0.0066 and *P* = 0.0337, respectively) and clinical decline (*P* = 0.0132; Fig. 4A and B), consistent with increased cachexia. Serum cytokines revealed elevated IL-1β and TNF-α in CB1R-KO mice (Fig. 4C and D), suggesting a heightened systemic inflammatory response. While TNF-α was also elevated in CB2R-KO mice, this did not reach significance. Notably, baseline TNF-α levels prior to infection were comparable across all genotypes, indicating that the cytokine elevations were infection-induced (Fig. 4C and D) . Survival analysis revealed that CB1R-KO mice were the most susceptible to infection, with only ~20% surviving until day 4 post-infection (*P* = 0.026 vs. WT; Fig. 4E). Despite similar clinical scores, CB2R-KO mice did not exhibit significantly increased mortality, suggesting that CB1R plays a more dominant role in regulating survival under high systemic bacterial burden. These results support a role for CB1R in limiting immunopathology and promoting survival during systemic infection, while CB2R may contribute more subtly to disease progression.

**Fig 5:**
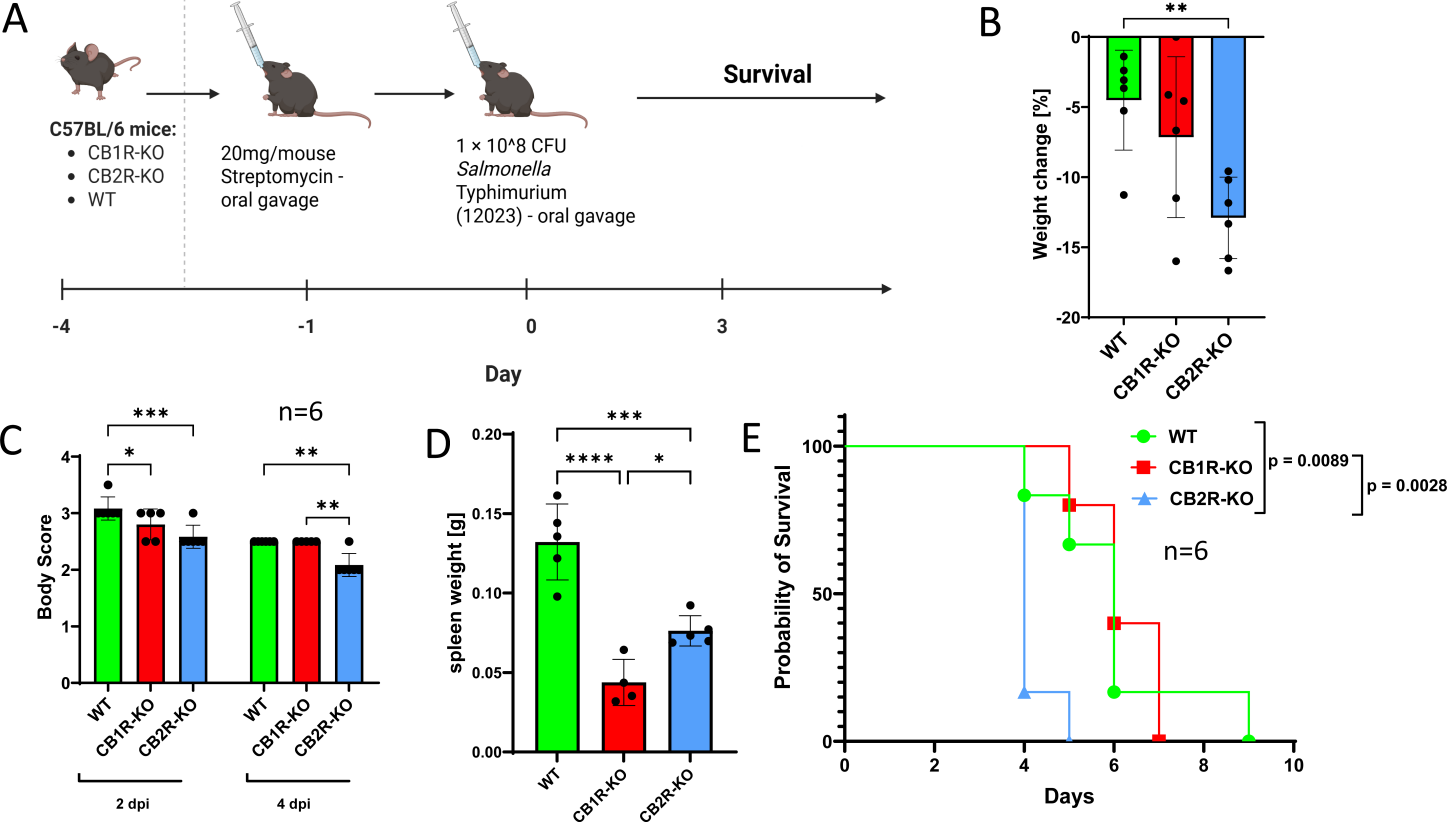
Role of CB1R and CB2R deficiencies in modulating clinical outcomes following *Salmonella* Typhimurium infection in streptomycin-pretreated C57BL/6 mice. (**A**) Schematic of the experimental setup: wild-type (WT), CB1 receptor knockout (CB1R-KO), and CB2 receptor knockout (CB2R-KO) C57BL/6 mice were pretreated with streptomycin followed by oral infection with 1 × 10⁸ CFU of *Salmonella* Typhimurium. (**B**) Percentage of body weight change at 2 days post-infection (DPI) in WT, CB1R-KO, and CB2R-KO mice. (**C**) Body condition scores at 2 and 4 dpi. (**D**) Spleen weights at 3 dpi. (**E**) Kaplan–Meier survival curves for WT, CB1R-KO, and CB2R-KO mice (*n* = 6 per group). Survival differences were analyzed using the log-rank (Mantel–Cox) test. *N* = 6. Data are presented as mean ± SEM. For panels **B**–**E**, statistical analysis was performed using one-way ANOVA. N values are indicated on figures as individual dots representing different experimental animals. Statistical significance is indicated as follows: *P < 0.05, *P < 0.01.

To evaluate CB2R function in mucosal infection, we used a colitis-associated model involving streptomycin pretreatment to facilitate intestinal colonization by *S*. Typhimurium (Fig. 5A). Under these conditions, CB2R-KO mice exhibited significantly greater weight loss (Fig. 5B) worsened body condition (Fig. 5C), and reduced survival (Fig. 5E) compared to WT controls. Both knockout groups had reduced spleen weights at 3 dpi (Fig. 5D). Importantly, only CB2R-KO mice displayed worsened survival in both systemic and mucosal infection models, whereas CB1R-KO mice showed significantly reduced survival only in systemic model of infection (Fig. 4E) but not in the mucosal infection model (Fig. 5E).

**Fig 6:**
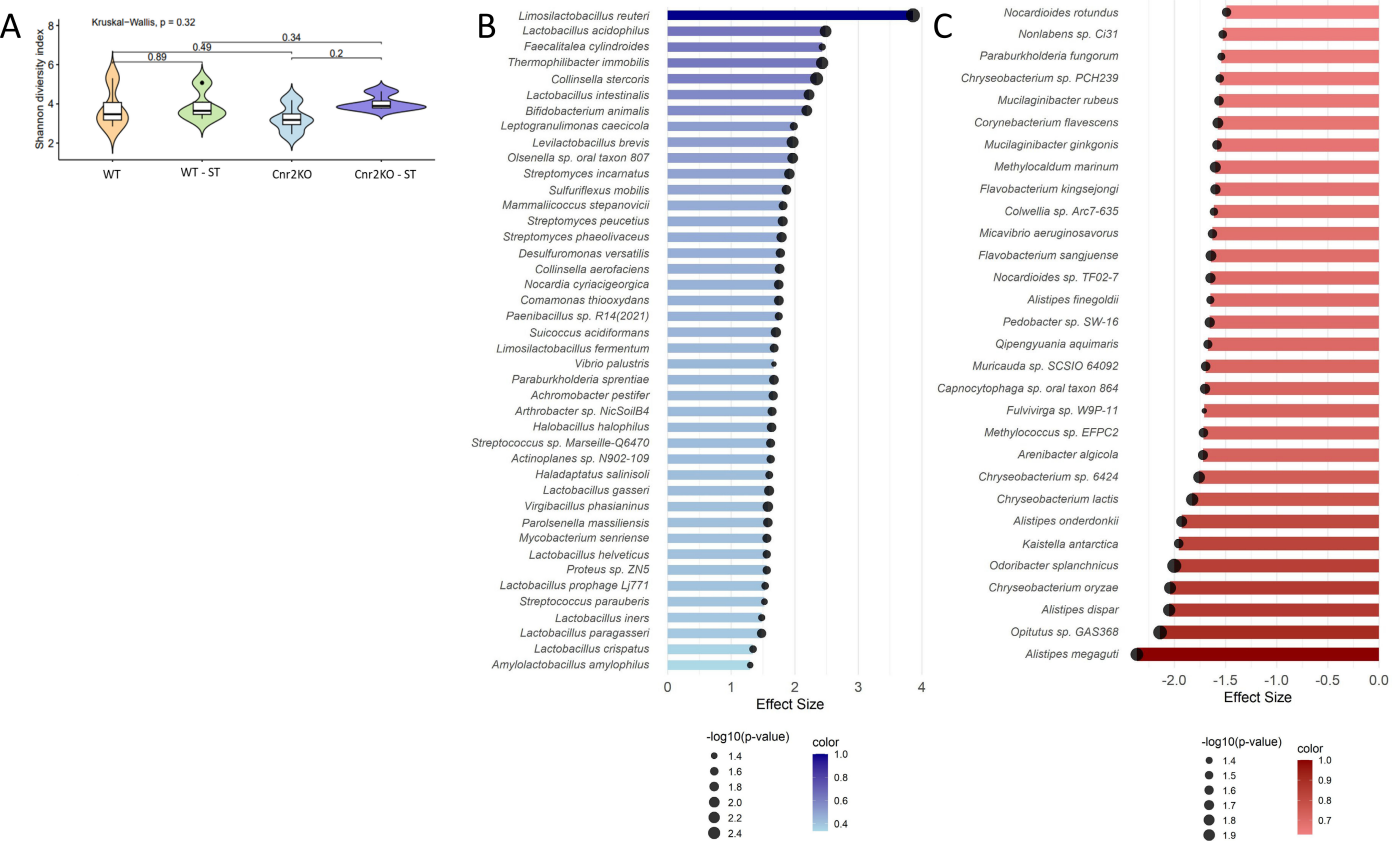
Gut microbiome alterations in CB2R-KO mice during *Salmonella* infection. (**A**) Alpha diversity of the gut microbiota in CB2R knockout (CB2R-KO) and wild-type (WT) littermate mice at baseline (pre-infection) and 4 days post-infection (dpi) with *Salmonella enterica* serovar Typhimurium. Diversity was assessed using the Shannon diversity index. Statistical significance was determined using the Kruskal–Wallis test. (**B and C**) Differential abundance analysis of microbial taxa 4 days post-infection. Blue bars indicate taxa enriched in WT; red bars indicate enrichment in CB2R-KO. Circle size represents −log10(*P*-value). Data were collected from littermate animals (*n* = 4 per group), housed in identical conditions with no more than five animals per cage.

Together, these data indicate that CB1R and CB2R deficiencies are associated with distinct patterns of disease susceptibility during *Salmonella* infection. CB1R deficiency is associated with elevated systemic inflammation and reduced survival following oral challenge, whereas CB2R deficiency is linked to worsened outcomes in both colitis and systemic model of salmonellosis.

### CB2R knockout mice display alterations in gut microbiome composition before and after *Salmonella* challenge

**Fig 7:**
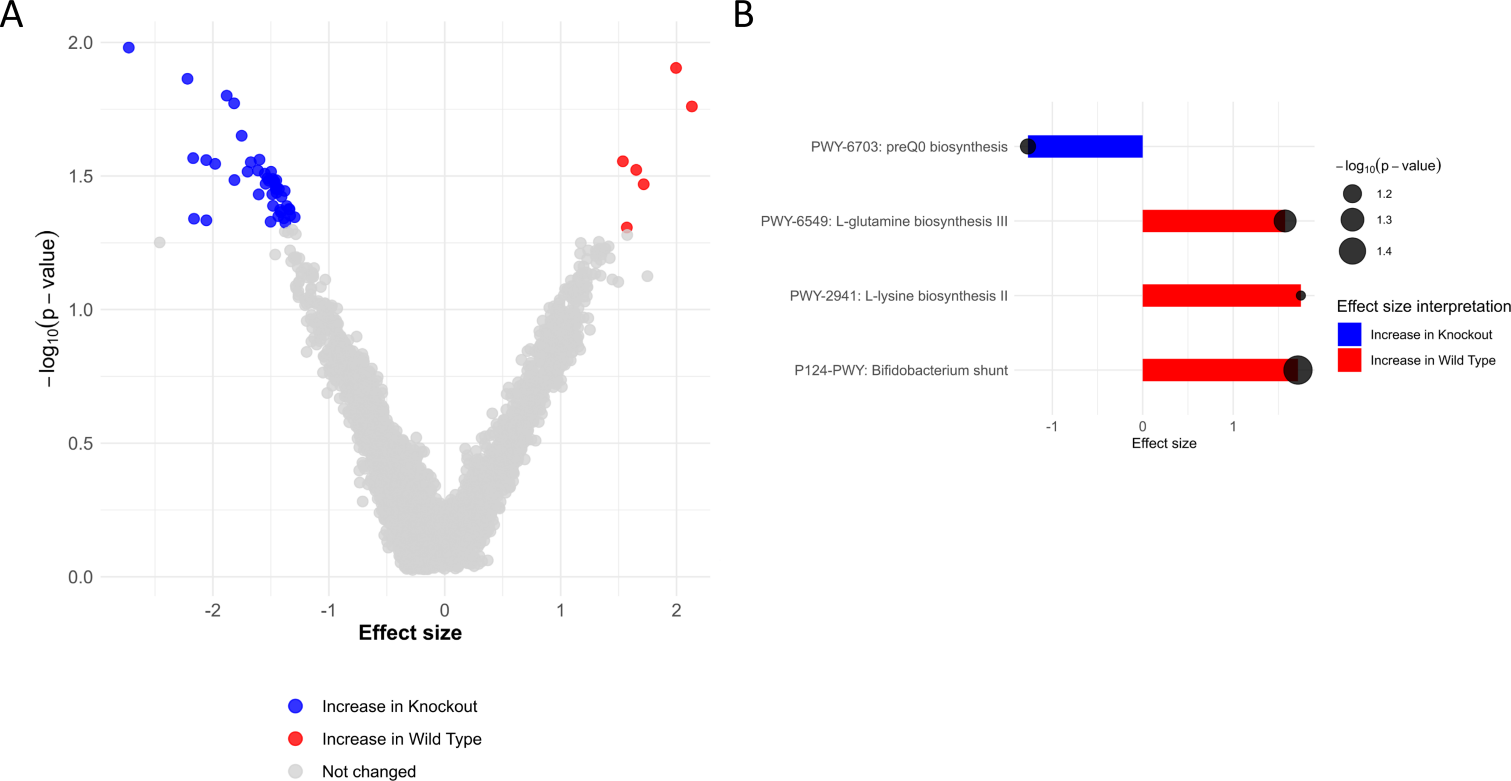
Functional microbiome analysis of CB2R-KO vs. WT mice post-infection. (**A**) The volcano plot displays differentially abundant metabolic pathways, with red indicating pathways enriched in WT and blue in CB2R-KO. (**B**) Bar plots highlight significantly altered pathways, including downregulation of *Bifidobacterium* shunt activity, L-glutamine, and L-lysine biosynthesis in CB2R-KO mice. Circle size reflects −log10(*P*-value). Data are derived from the analysis presented in Fig. 6.

CB2R-KO mice exhibited worsened clinical outcomes following *Salmonella* Typhimurium infection, with the most pronounced effects observed in the colitis-associated model. These differences occurred despite no significant variation in systemic bacterial burden or macrophage intracellular infection compared to WT mice, suggesting that immune cell-intrinsic bacterial control mechanisms were not solely responsible for the heightened susceptibility. Given the well-established role of the gut microbiota in regulating mucosal immunity and resistance to enteric pathogens (38–42), we assessed whether CB2R deficiency altered gut microbial composition. Whole-genome shotgun metagenomic sequencing of fecal samples from CB2R-KO and WT mice was performed at baseline and after infection. While overall alpha diversity (Shannon index) was not significantly different between genotypes (*P* = 0.32; Fig. 6A), distinct differences in species composition were observed. Prior to infection, microbial profiles were broadly similar between CB2R-KO and WT mice (Fig. S6 and S7; Table S1), although there were several microbes increased in CB2R-KO mice compared to WT at baseline, such as an increase in certain *Clostridia* and decrease in *Lacticaseibacillus casei* (Fig. S7). There were, however, more changes to the microbiome after infection. Specifically, CB2R-KO mice had significantly reduced abundance of several beneficial bacterial species, including *Lactobacillus acidophilus*, *L. intestinalis*, *L. gasseri*, *L. crispatus*, and *Bifidobacterium animalis* (Fig. 6B; Table S2). These taxa have been previously associated with gut barrier support, immune modulation, and colonization resistance. In contrast, CB2R-KO mice showed increased relative abundance of *Alistipes* species (*A. humii*, *A. finegoldii*, and *A. onderdonkii*) (Fig. 6C; Table S2), which have been linked to both homeostatic and pro-inflammatory outcomes in other contexts. Functional analysis of microbial communities during infection revealed significant alterations in predicted metabolic pathways in fecal specimens of CB2R-KO mice. Specifically, there was a reduction in several key pathways, including the Bifidobacterium shunt, L-glutamine biosynthesis III, and L-lysine biosynthesis II, as well as an increase in preQ₀ biosynthesis (Fig. 7).

Overall, although these analyses do not establish a direct causal relationship, the results indicate that CB2R deficiency is associated with both compositional and functional changes of the gut microbiota during *Salmonella* infection.

## DISCUSSION

Effective host defense against *Salmonella* requires a finely balanced immune response—insufficient control leads to bacterial dissemination, while excessive inflammation causes tissue damage and worsened disease outcomes (43). Here, we demonstrate that the cannabinoid receptors CB1R and CB2R play non-redundant roles in shaping the immune response during *Salmonella* challenge. Using both systemic and colitis-associated infection models, we show that CB1R predominantly constrains systemic inflammation and promotes host survival, while CB2R is critical for maintaining mucosal immune balance and microbial homeostasis. Although both CB1R- and CB2R-deficient mice exhibited increased susceptibility under high-burden infection, their underlying immune phenotypes and tissue-specific vulnerabilities diverged significantly.

Unexpectedly, loss of CB1R had a pronounced impact on systemic immune dynamics and survival. While CB1R is widely studied for its roles in the central nervous system (44, 45), it is also expressed in immune cells such as macrophages (46, 47), although much less studied than CB2R. CB1R modulates inflammatory responses through activation by endogenous ligands (AEA, 2-AG) or synthetic agonists such as WIN 55,212-2 (46, 47). CB1R signaling inhibits adenylate cyclase activity and modulates ion channel function (48–52), with downstream effects including reduced cytokine production and suppression of inflammasome activation (13, 51, 52). In our study, CB1R-deficient mice displayed elevated systemic levels of IL-1β and TNF-α, increased bacterial burdens, and reduced survival, consistent with a failure to control inflammation and pathogen spread. At the cellular level, CB1R-deficient macrophages adopted a skewed M2b phenotype, characterized by high expression of TNF-α, IL-6, and TGF-β alongside reduced IL-10. Although M2b macrophages are often linked to immunoregulatory functions, this specific cytokine profile may support persistent, low-grade inflammation while impairing antimicrobial defenses. Notably, reduced IL-10 and elevated TGF-β have been associated with diminished nitric oxide production—an important mechanism for intracellular *Salmonella* clearance (53). This apparent paradox—systemic hyperinflammation occurring alongside macrophage-mediated immunosuppression—illustrates the complication of macrophage polarization states and their context-dependent roles in infection (28, 54, 55). Inflammatory responses, while initially beneficial, can become maladaptive in the absence of regulatory cytokines (56). Importantly, while TNF-α and IL-10 are central to immune regulation, our data suggest that susceptibility in CB1R-deficient mice cannot be attributed to these cytokines alone. It likely reflects a broader immune imbalance involving cell recruitment, cell polarization, and possibly some compensatory mechanisms.

CB1R deficiency also impaired neutrophil recruitment to infected tissues. Although CB1R’s role in neutrophil biology is less well understood, previous studies suggest it supports chemotaxis and trafficking (57). Our findings indicate that CB1R loss may indeed disrupt these processes, contributing to the dysregulated macrophage–neutrophil dynamics that are associated with poor infection outcomes (38). Interestingly, CB1R deficiency did not exacerbate disease severity in the colitis-associated model, suggesting that its protective effects are more pronounced in systemic compartments. Redundant signaling pathways or localized compensatory mechanisms may buffer CB1R loss in this salmonellosis model.

In contrast to CB1R, CB2R—predominantly expressed in peripheral immune tissues—plays a well-established role in promoting anti-inflammatory macrophage polarization and resolving inflammation (9, 10, 12, 13, 58). In our study, in CB2R-deficient mice, macrophages adopted a pro-inflammatory M1a phenotype, with elevated IL-6 and reduced IL-10 levels. Despite increased neutrophil infiltration, bacterial clearance was not enhanced and was moderately impaired, suggesting that excessive neutrophilia contributed to immunopathology rather than improved control of infection. These results are consistent with prior findings that CB2R signaling dampens neutrophil-driven inflammation (58). CB2R deficiency was particularly detrimental in the streptomycin-pretreated colitis model, where mice exhibited accelerated clinical decline, rapid weight loss, and earlier mortality compared to both wild-type and CB1R-deficient animals. Given that streptomycin disrupts the gut microbiota, these findings suggest that CB2R’s protective role may be, at least in part, microbiota-independent. Alternatively, the absence of protective commensals and neutrophilia may amplify CB2R-mediated immune dysfunction at mucosal sites. These results prompt the need for microbiota-controlled studies to better understand how CB2R signaling contributes to mucosal immune resilience.

Given prior reports linking the endocannabinoid system to gut microbial dynamics (59), we investigated if CB2R deficiency influenced microbiota composition during infection. Metagenomic sequencing revealed that CB2R-KO mice had reduced relative abundance of beneficial commensals—such as *Limosilactobacillus reuteri*, *L. acidophilus*, *L. brevis*, and *Bifidobacterium animalis*—which are known to support gut barrier function and suppress inflammation (60, 61). The loss of these protective microbes may compromise gut homeostasis, heighten inflammation, and impair immune tolerance (39–41). In contrast, *Alistipes* species were enriched, although their functional roles in inflammation are complex and may play both beneficial and pro-inflammatory functions (40, 62, 63). Functional pathway analysis of metagenomic data suggested reduced relative abundance of microbial genes involved in short-chain fatty acid (SCFA) metabolism and amino acid biosynthesis in CB2R-deficient mice. Predicted pathways, such as the Bifidobacterium shunt and L-glutamine biosynthesis, have been implicated in barrier repair and immune modulation (64–68). However, these findings remain predictive and must be interpreted cautiously, as they are not yet supported by transcriptomic or metabolomic validation. Overall, it is important to emphasize that while CB2R deficiency was associated with altered microbial composition, we cannot infer causality. The observed dysbiosis may be a consequence of inflammation and disease progression rather than a predisposing factor for worsening outcomes observed. Future studies using fecal microbiota transplantation or germ-free mouse models will be required to disentangle cause from consequence.

Therapeutically, enhancing CB1R signaling could help mitigate cytokine-driven pathology in systemic infections. However, prior studies have shown that CB1R agonism may also exacerbate inflammation in different infectious disease contexts. For example, Δ9-THC increased pro-inflammatory cytokine production during *Legionella pneumophila* infection (69), while CB1R antagonism enhanced macrophage antimicrobial function in *Brucella suis* infection (70). These findings show the need to carefully calibrate CB1R-targeted therapies in the future to avoid impairing protective immunity against other pathogens. Because CB1R and CB2R are expressed in other cell types other than macrophages (such as described in B cells, T cells, and dendritic cells) that exist within splenic tissues (71–74), future studies comparing their cell-specific expression of these receptors between mice and humans are further warranted.

By contrast, CB2R signaling has demonstrated more consistent protective effects across inflammatory models. In our study, CB2R deficiency was associated with mucosal immune dysregulation, neutrophil overactivation, and infection-associated microbiota alterations. Beyond infectious models, CB2R activation has also been shown to protect against chemically induced colitis (75). CB2R agonists have been shown to inhibit neutrophil infiltration, NF-κB signaling, and NLRP3 inflammasome activation (58). These properties position CB2R as a promising therapeutic target to restore mucosal immune homeostasis and mitigate infection-associated pathology, particularly in enteric diseases where inflammation and microbiota disruption are intertwined.

### Conclusions

Our findings reveal distinct and complementary roles for CB1R and CB2R in shaping immune responses to *Salmonella* infection. CB1R primarily limits systemic inflammation and promotes coordinated macrophage–neutrophil responses necessary for bacterial clearance and host survival. In contrast, CB2R contributes to mucosal immune equilibrium and microbiota stability, and its presence seemed to be particularly critical in the context of colitis-associated infection. CB2R deficiency led to pro-inflammatory macrophage polarization, excessive neutrophil recruitment, and infection-driven dysbiosis and was associated with increased mortality in both systemic and mucosal models. Collectively, these results uncover the importance of endocannabinoid signaling in balancing protective versus pathological immunity during bacterial challenge. While more mechanistic studies and effect on other pathogens are needed, our findings suggest that selective targeting of CB1R and CB2R may offer therapeutic avenues to fine-tune immune responses in infectious disease.

## MATERIALS AND METHODS

### Bacterial strains

*Salmonella enterica* serovar Typhimurium strain ATCC 14028 was used for murine studies, and *Salmonella* Typhimurium strain UK-1 was used for cell infections. Additionally, a nalidixic acid (Nal)-resistant derivative of *S.* Typhimurium wild-type isolate ATCC 14028 (76) was used in selected experiments to facilitate quantification of bacterial burden on selective agar plates. For murine studies, bacteria were cultured in Luria-Bertani (LB) Lennox broth at 37°C with shaking until they reached mid-log growth (OD_600_ = 0.75). For cell culture infections, the bacteria were cultured in LB Lennox broth at 37°C with shaking until they reached OD_600_ = 0.5. Bacteria were then harvested, washed with phosphate-buffered saline (PBS), and resuspended in PBS to the appropriate concentration for infection.

### Animal breeding

CB1 receptor knockout (CB1R-KO) and CB2 receptor knockout (CB2R-KO) mice on a C57BL/6 background were used for all experiments (77, 78). Mice were housed under specific pathogen-free conditions with *ad libitum* access to food and water. Mice were age and sex matched and maintained under identical environmental conditions throughout the study. Littermates were housed together. Males and females were cohoused only from birth until weaning. Knockout and wild-type (WT) mice were cohoused after weaning when they originated from the same litter, in order to preserve consistent early-life microbial exposure. Four or five mice were housed per cage to minimize stress and environmental variability. Genotypes were confirmed by PCR using DNA extracted from ear punches. Mice were identified using ear-notch coding at weaning. All genotyping was conducted by personnel blinded to behavioral group assignments. Animals were monitored daily for general health. Any mice exhibiting signs of distress or illness were evaluated by veterinary staff and removed from the study if deemed necessary.

### *Salmonella* infection in mice

Male and female C57BL/6 mice (8–12 weeks old) from the holding colony were randomly assigned to infection groups. When possible, littermate controls were used to minimize microbiota-related variations in *Salmonella* colonization. Mice were orally infected with *Salmonella enterica* serovar Typhimurium (7.5 × 10⁷ CFU) in 50 µL of sterile PBS using a gavage needle, while control mice received PBS alone.

For the mucosal infection model, which enhances susceptibility to colonic colonization and inflammation, a previously described protocol was followed (79). Mice were pretreated with oral streptomycin (20 mg/mouse in sterile water). Twenty-four hours later, mice received 100 µL of 0.2 M sodium bicarbonate by oral gavage to transiently neutralize gastric acidity. Ten minutes after bicarbonate administration, mice were orally challenged with *S*. Typhimurium (1 × 10⁸ CFU) in 100 µL of sterile PBS. Control animals received PBS alone following the same pretreatment regimen.

Infections were monitored post-infection. Body weight and condition scores were recorded daily, and serum cytokine levels were measured to assess systemic inflammatory responses. Mice were observed daily for clinical signs of infection, including weight loss and overall body condition, using a standardized scoring system. Both weight and body condition scores were recorded daily for up to 14 days post-infection. To assess survival, mice were monitored for 14 days, and survival rates were recorded each day. Mice exhibiting severe clinical symptoms were euthanized in accordance with IACUC guidelines.

Survival data were analyzed using the Kaplan–Meier method, and statistical significance was determined using the log-rank (Mantel–Cox) test. For experiments requiring tissue analysis, mice were euthanized at 4 days post-infection. Spleen and liver tissues were aseptically collected, homogenized in sterile PBS using a TissueLyser LT (Qiagen), and subjected to serial dilution. Dilutions were plated on LB agar and incubated at 37°C for 24 hours. Colony-forming units (CFU) were then counted to quantify bacterial burden in each organ.

### Flow cytometry

Spleens were collected from both infected and control mice, and single-cell suspensions were prepared by mechanically dissociating the tissues through a 70 µm cell strainer. Red blood cells were lysed using RBC Lysis Buffer (Invitrogen), and the remaining cells were incubated with a Live/Dead viability dye (Zombie Aqua) at 4°C for 15 minutes. After washing with FACS buffer (PBS containing 1% BSA), the cells were treated with Fc Block (TruStain fcX anti-mouse CD16/32, BioLegend) for 5 minutes to minimize non-specific binding. Surface marker staining was performed by incubating the cells with an antibody cocktail targeting specific surface markers at 4°C for 30 minutes. Following another wash with FACS buffer, cells were fixed with Cytofix/Cytoperm solution (BD Biosciences) for 15 minutes. Fixed cells were washed twice with Perm/Wash buffer (BD Biosciences) before staining for intracellular markers. Flow cytometric data were acquired using the CytoFLEX flow cytometer (Beckman Coulter) and analyzed using FlowJo software (Tree Star, Inc.).

Flow cytometry was used to characterize immune cell populations and their functional states in spleens from infected and control mice using multiple staining panels. For the identification of lymphoid and myeloid cell populations, single-cell suspensions were stained with CD45 (Pacific Blue, Cat# 157212) as a leukocyte marker, Zombie Aqua (Cat#77143) for viability, CD3 (PerCP/Cy5.5, Cat# 100217) for T cells, CD19 (APC/Fire750, Cat# 115557) for B cells, CD11b (Alexa Fluor 647, Cat# 101218) for myeloid cells, NK1.1 (Alexa Fluor 700, Cat# 156511) for natural killer (NK) cells, F4/80 (PE, Cat# 123109) for macrophages, and Ly6G (Alexa Fluor 488, Cat# 127625) for neutrophils. A second panel was used to evaluate the activation and polarization of myeloid cells, incorporating CD86 (Pacific Blue, Cat# 105021) as an activation marker for antigen-presenting cells and CD206 (Alexa Fluor 700, Cat# 141733) for M2 macrophages, alongside the same markers for other immune subsets. A separate panel was designed to detect intracellular *Salmonella*, using CD45 and CD11b to gate on leukocytes and myeloid cells, F4/80 to identify macrophages, and a FITC-conjugated *Salmonella*-specific antibody (Invitrogen, Ref # PA1-73020) to detect intracellular bacteria. Cytokine production was assessed using a panel that included IL-6 (PE, Cat# 504504) as pro-inflammatory markers, and IL-10 (Alexa Fluor 488, Cat# 505013) and TGF-β1 (PerCP/Cy5.5, Cat# 141410) as anti-inflammatory markers, along with CD45, CD11b, and F4/80 to identify macrophages. All antibodies were purchased from BioLegend, except for the *Salmonella*-specific antibody (Invitrogen).

### Cytokine measurements

Blood was collected from both infected and control mice at the indicated time points via saphenous vein bleeding. Blood samples were centrifuged at 10,000 × *g* for 10 minutes at 4°C to separate the serum. The samples were then stored in −20C until further use. The levels of TNF-α and IL-1β in the serum were quantified using specific ELISA kits (R&D Biosystems) according to the manufacturer’s instructions.

### BMDM cell culture and infection

Bone marrow-derived macrophages (BMDMs) were generated from mesenchymal stem cells isolated from the hindlimbs of wild-type C57BL/6 mice. Cells were cultured in RPMI 1640 supplemented with macrophage colony-stimulating factor (M-CSF) (25 ng/mL) to promote differentiation into macrophages. The culture medium, including M-CSF, was refreshed every three days, and BMDMs were considered mature on day 7. For infections, BMDMs were seeded at 6 × 10⁵ cells per well in 12-well plates and allowed to adhere for 24 hours before infection. *Salmonella enterica* serovar Typhimurium strain UK-1 was grown overnight in Lennox LB broth (16 hours, 37°C, shaking). A subculture was established in fresh Lennox LB broth (25 mL) and grown to an optical density (OD_600_) of 0.5 to ensure bacteria were in the mid-logarithmic growth phase. BMDMs were infected with *Salmonella* at an MOI of 10 in incomplete RPMI 1640 for 1 hour. Following infection, cells were washed with PBS and incubated in RPMI supplemented with gentamicin (100 µg/mL) for 1 hour to eliminate extracellular bacteria. The medium was then replaced with RPMI containing gentamicin (25 µg/mL), and cells were incubated until designated time points. Cell pellets were stored in RNAlater for downstream analyses.

### Microbiome analysis

Fecal samples were collected from CB2R knockout (CB2R-KO) and wild-type (WT) littermate mice (*n* = 4 per group) housed under identical conditions. Animals were co-housed, with no more than five mice per cage, to control for cage effects on microbial composition. Stool collection was performed the day before infection (baseline) and at 4 days post-infection. Mice were placed in sterile containers for stool collection, which was performed the day before infection (baseline) and 4 days post-infection. Stool samples were collected using sterile tools and immediately transferred into sterile Transnetyx tubes containing DNA stabilization buffer. Samples were temporarily stored at 4°C before being shipped under controlled conditions to Transnetyx for processing the following day. Microbiota profiling was conducted using shallow shotgun whole-genome sequencing on an Illumina platform (1 × 150 bp). Raw sequencing reads were processed to remove low-quality sequences (expected error >0.5) and fragments shorter than 150 bp using Vsearch (80). High-quality sequences were then taxonomically classified using Kraken 2 (81) with the standard database. The resulting contingency table was converted into a phyloseq object for downstream analyses (82). To ensure uniform sequencing depth across samples, data were rarefied to a minimum library size of 1,609,000 reads per sample. Alpha diversity metrics were calculated using the microbiome package in R. Boxplots summarizing alpha diversity distributions were generated using ggplot2^71^, and statistical significance was assessed using the Kruskal–Wallis test from base R. Differential abundance analysis was performed using the ALDEx2 package (83). Sequence files were processed to remove low-quality sequences (expected error >0.5) and sequences smaller than 150 bp using Vsearch. The remaining high-quality sequences were classified using Kraken 2 and the standard database. The resulting contingency table was converted into a phyloseq object for downstream analyses. Data were rarefied by the minimum library size of 1,609,000 per sample. Alpha diversity was measured by using the rarefied data set with the microbiome package. Boxplots summarizing the alpha diversity distribution were plotted by using ggplot2 R package. The significance of numerical evaluations of alpha diversity was tested using the Kruskal–Wallis test from base R. The ALDEx2 package was used to calculate differential abundance. Functional microbiome analysis was conducted with HUMAnN 3.0 to identify shifts in metabolic pathways (84).

### qPCR analysis

Cells were collected via cell scraping, resuspended in RNAlater (Thermo Fisher Scientific), and stored at –20°C until further processing. Total RNA was extracted from cell pellets using the RNeasy Mini Kit (Qiagen), following the manufacturer’s instructions. RNA concentration and purity were assessed using a NanoDrop spectrophotometer (Thermo Fisher Scientific). Complementary DNA (cDNA) was synthesized from 1 µg of total RNA using the iScript Reverse Transcription Supermix for RT-qPCR (Bio-Rad). Quantitative real-time PCR (RT-qPCR) was performed using SsoAdvanced Universal SYBR Green Supermix (Bio-Rad) on a CFX96 Real-Time System (Biorad). Primer sequences for *cnr1* (Cat #10025636, qMmuCEP0038879) and *cnr2* (Cat #10041595, qMmuCEP0039299) were obtained from the PrimePCR SYBR Green Assay (Bio-Rad). Primers for assessing *arg1* (M_Arg1_1_NM_007482), *tnfa* (M_Tnf1_1_NM_013693), *tlr4* (M_Tlr4_1_NM_021297), and *nos2* (M_Nos2_1_NM_01-0927) expression were purchased from Eton Biosciences or Sigma-Aldrich. Melt-curve analysis was conducted to verify primer specificity and amplification efficiency. Relative gene expression was calculated using the ΔΔCt method, with β-actin as the internal reference control.

### Statistical analysis

Data are presented as mean ± SEM. Statistical significance between groups was evaluated using Student’s *t*-test or one-way ANOVA followed by Tukey’s *post hoc* test for multiple comparisons, as appropriate. A *P*-value of <0.05 was considered statistically significant.

## Data Availability

All data supporting the findings of this study are included in this article and the supplemental material. The full microbiome data set has been deposited in Mendeley Data (ver. 2) under DOI:10.17632/7pn4pwj3td.1. A preprint of the manuscript is available on bioRxiv (85).

## SUPPLEMENTAL MATERIAL

Supplemental material mbio.02088-25-s0001.docx Supplemental figures and tables.

ASM does not own the copyrights to Supplemental Material that may be linked to, or accessed through, an article. The authors have granted ASM a non-exclusive, world-wide license to publish the Supplemental Material files. Please contact the corresponding author directly for reuse.
