# Supplementary material for: Deficiency of cannabinoid receptors enhances host susceptibility to bacterial infection
Source: mBio. 2025 Sep 18;16(10):e02088-25. doi: 10.1128/mbio.02088-25 (PMC12506127; doi:10.1128/mbio.02088-25)
Supplement: Supplemental material — Supplemental figures and tables. [file mbio.02088-25-s0001.docx]

**Supplementary Figures**


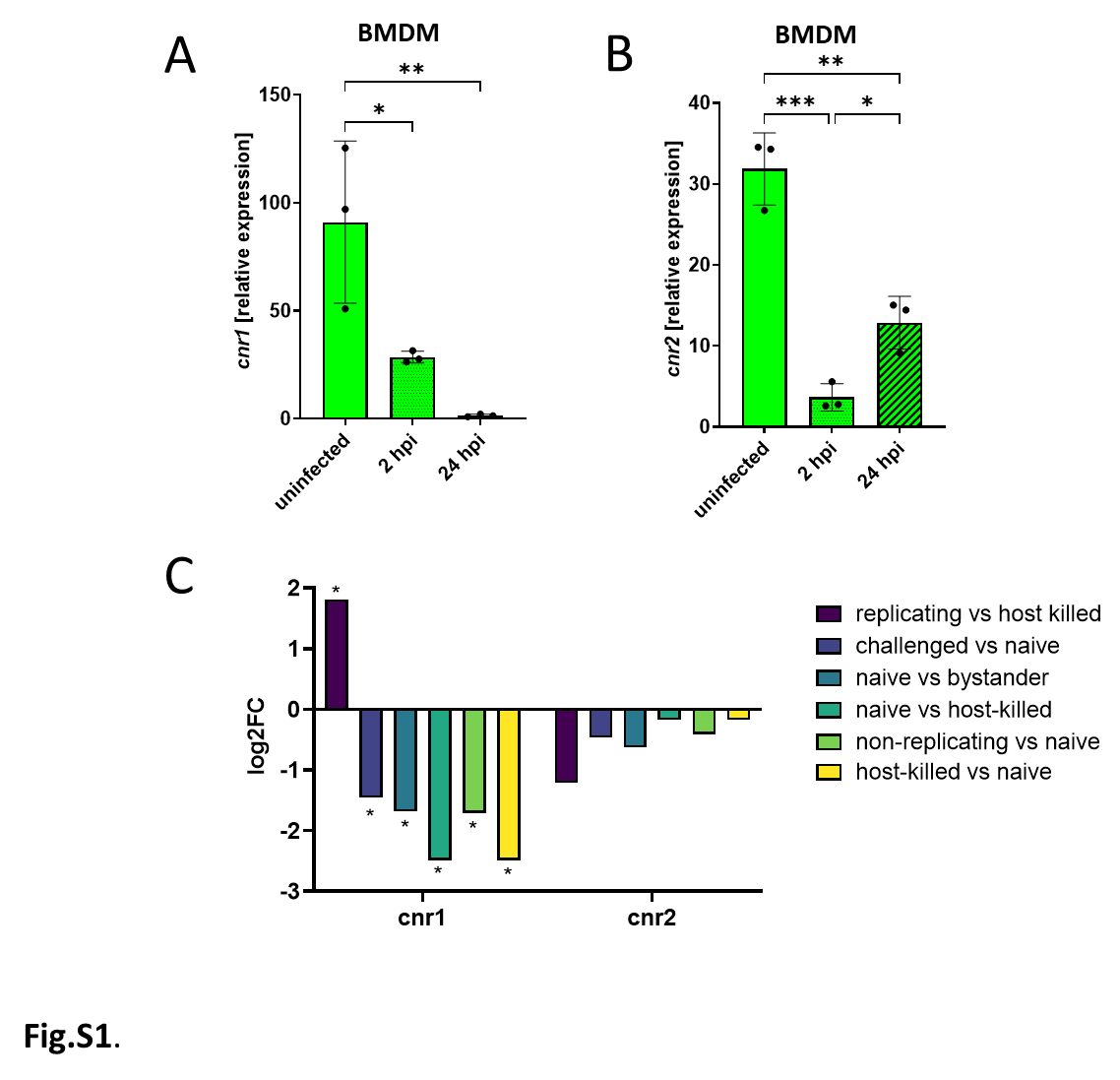


**Figure S1. (A, B) Cnr1 and Cnr2 expression in *Salmonella*-infected macrophages over time.** The gene expression levels of *Cnr1* and *Cnr2* were analyzed by qPCR in BMDMs following *Salmonella* infection with an MOI of 10. Samples were collected at 0-, 2-, and 24-hours post-infection (hpi). Expression was normalized to beta-actin housekeeping gene. Data represent the mean ± SEM from three biological replicates. Statistical significance is indicated as follows: *p < 0.05, **p < 0.01, ***p < 0.001. **(C).** Differential expression analysis of CNR1 and CNR2 was performed using RNA-seq data from Schade *et al.* ^37^, which profiled transcriptomes of human THP-1 macrophages infected with *Salmonella* Typhi 18-h post-infection. Cannabinoid receptor expression across five macrophage populations: naïve, bystander, host-killed, non-replicating, and replicating is shown. Statistical significance was determined by adjusted p-values; asterisks indicate significant differential expression (**p* < 0.05).


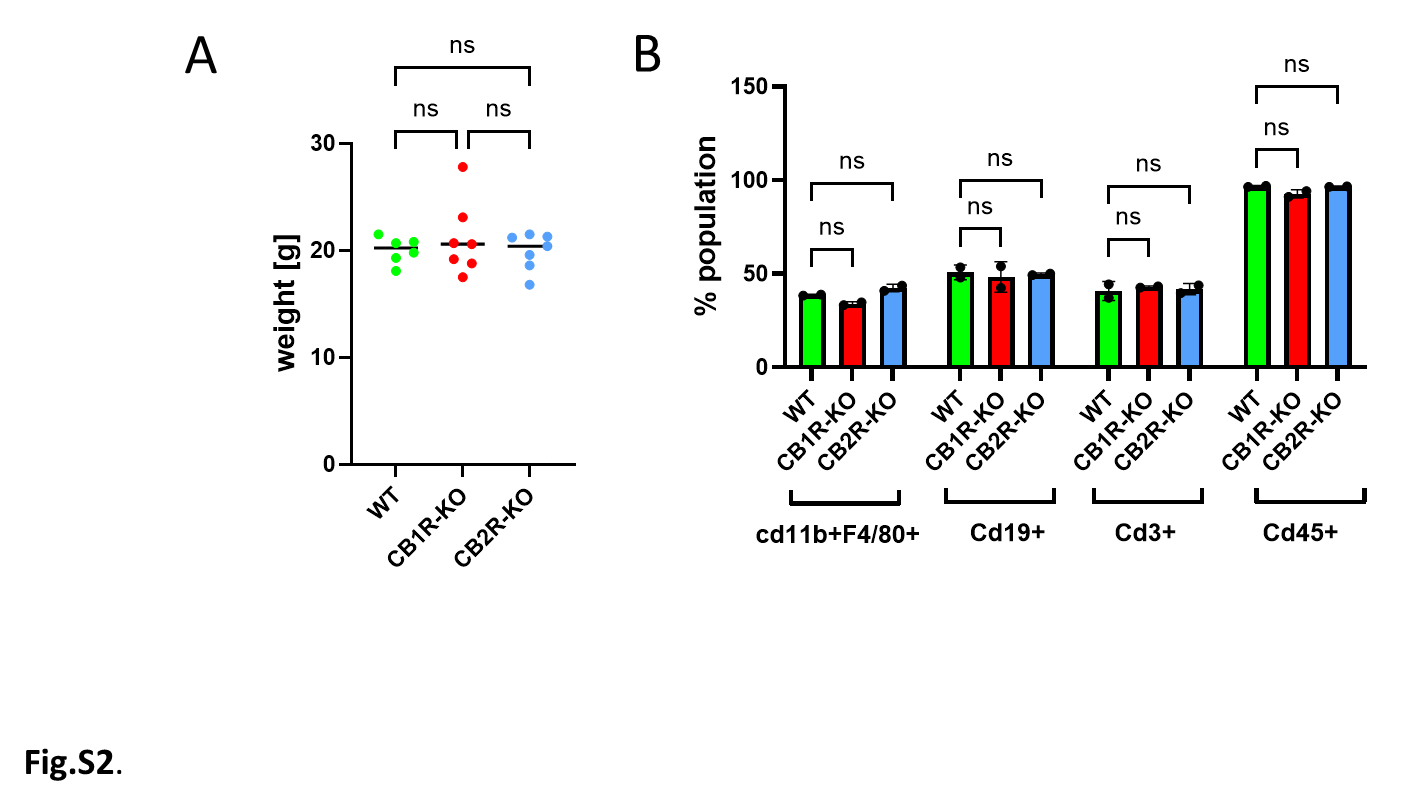


**Figure S2. Baseline characteristics of WT, CB1R-KO, and CB2R-KO mice prior to *Salmonella* Typhimurium infection. (A)** Body weights of wild-type (WT), CB1 receptor knockout (CB1R-KO), and CB2 receptor knockout (CB2R-KO) C57BL/6 mice prior to infection (n = 6 or 7 per group). **(B)** Flow cytometry quantification of live immune cell subsets in the spleen before infection, including CD11b⁺F4/80⁺ (macrophages), CD45⁺ (leukocytes), CD19⁺ (B cells), and CD3⁺ (T cells) (n = 2 per group).


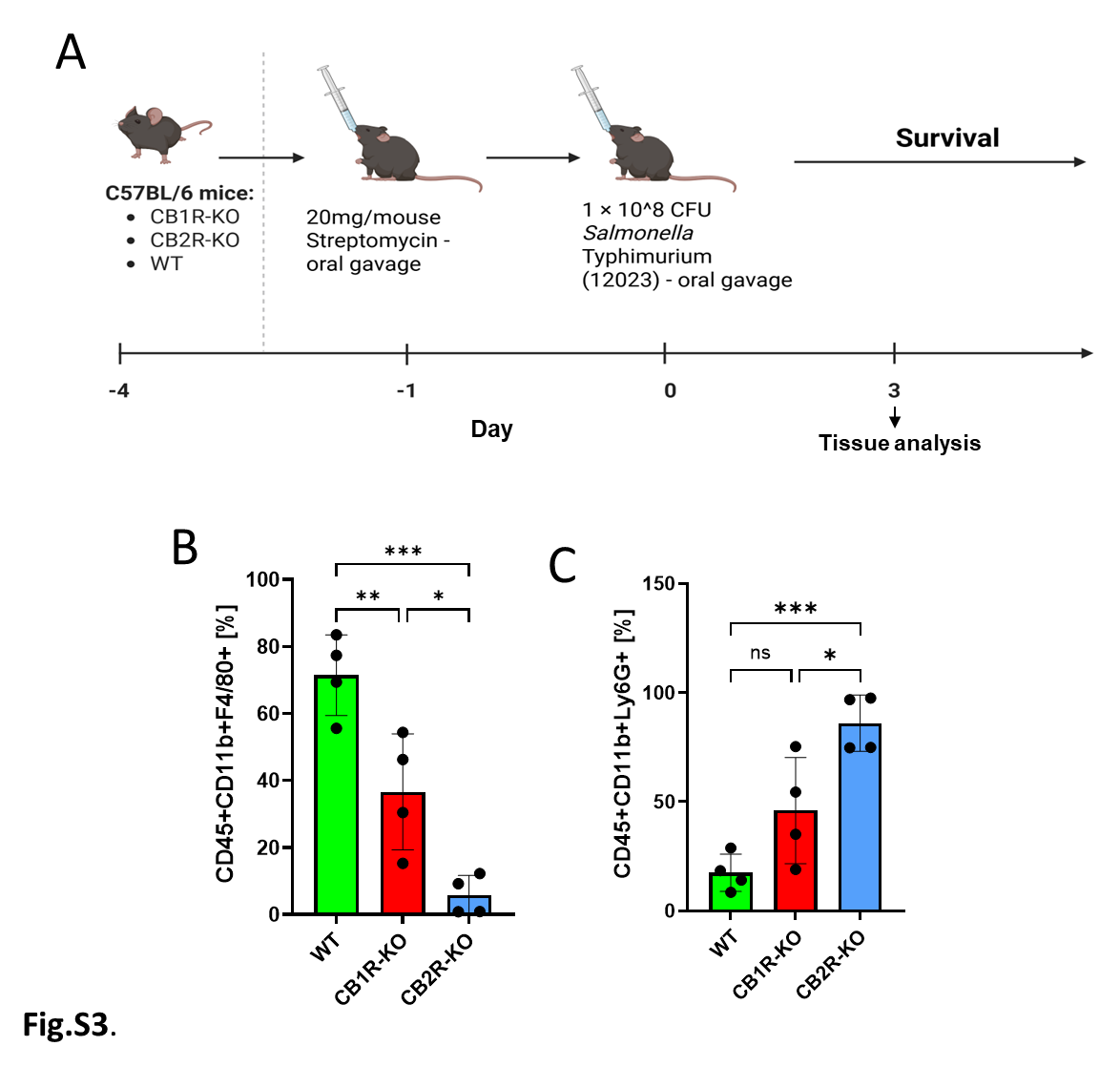


**Figure S3. CB1R and CB2R modulate splenic innate immune responses and cytokine expression following *Salmonella* Typhimurium infection. (A)** Experimental timeline for infection and analysis. C57BL/6 mice—wild-type (WT), CB1 receptor knockout (CB1R-KO), and CB2 receptor knockout (CB2R-KO)—were pre-treated with 20 mg/mouse streptomycin by oral gavage (day -1), followed by oral gavage with 1 × 10⁸ CFU *Salmonella* Typhimurium (strain 12023) on day 0. Survival was recorded daily. **(B-C)** Flow cytometry quantification of innate immune cell subsets in spleens 3 days post-infection: **(B)** Macrophages (Live CD45⁺CD11b⁺F4/80⁺) and **(C)** neutrophils (Live CD45+CD11b+Ly6G+). Data are presented as mean ± SEM, with individual data points representing single animals. A single cohort of littermate-controlled animals was used. Statistical comparisons were performed using one-way ANOVA followed by Tukey’s post hoc test. Significance levels: *p < 0.05, **p < 0.01, ***p < 0.001.


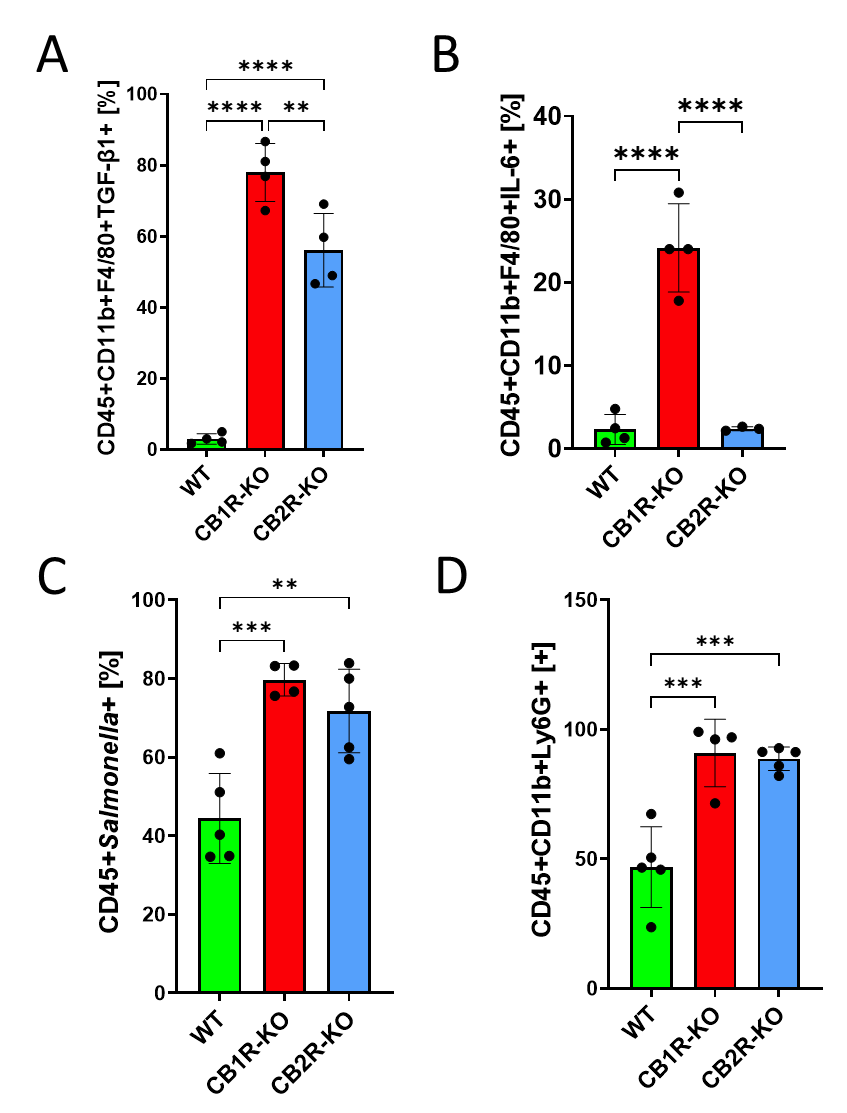


**Figure S4. CB1R and CB2R influence splenic cytokine expression and systemic inflammation in a colitis-associated model of *Salmonella* infection.** Wild-type (WT), CB1 receptor knockout (CB1R-KO), and CB2 receptor knockout (CB2R-KO) C57BL/6 mice were pretreated with streptomycin (20 mg/mouse, oral gavage) on day −1 and infected orally with *Salmonella enterica* serovar Typhimurium (1 × 10⁸ CFU, strain 12023) on day 0. **(A–B)** Intracellular cytokine staining of splenic macrophages (CD45⁺CD11b⁺F4/80⁺) at 3 days post-infection (dpi), showing expression levels of **(A)** TGF-β and **(B**) IL-6 across genotypes. **(C)** Flow cytometry analysis of peripheral blood leukocytes at 2 dpi, quantifying the percentage of *Salmonella*-positive live cells. **(D)** Frequency of circulating neutrophils (CD45⁺CD11b⁺Ly6G⁺) in peripheral blood at 2 dpi. Data are presented as mean ± SEM, with individual data points representing single animals. A single cohort of littermate-controlled animals was used. Statistical comparisons were performed using one-way ANOVA followed by Tukey’s post hoc test. Significance levels: *p < 0.05, **p < 0.01, ***p < 0.001, ****p < 0.0001.


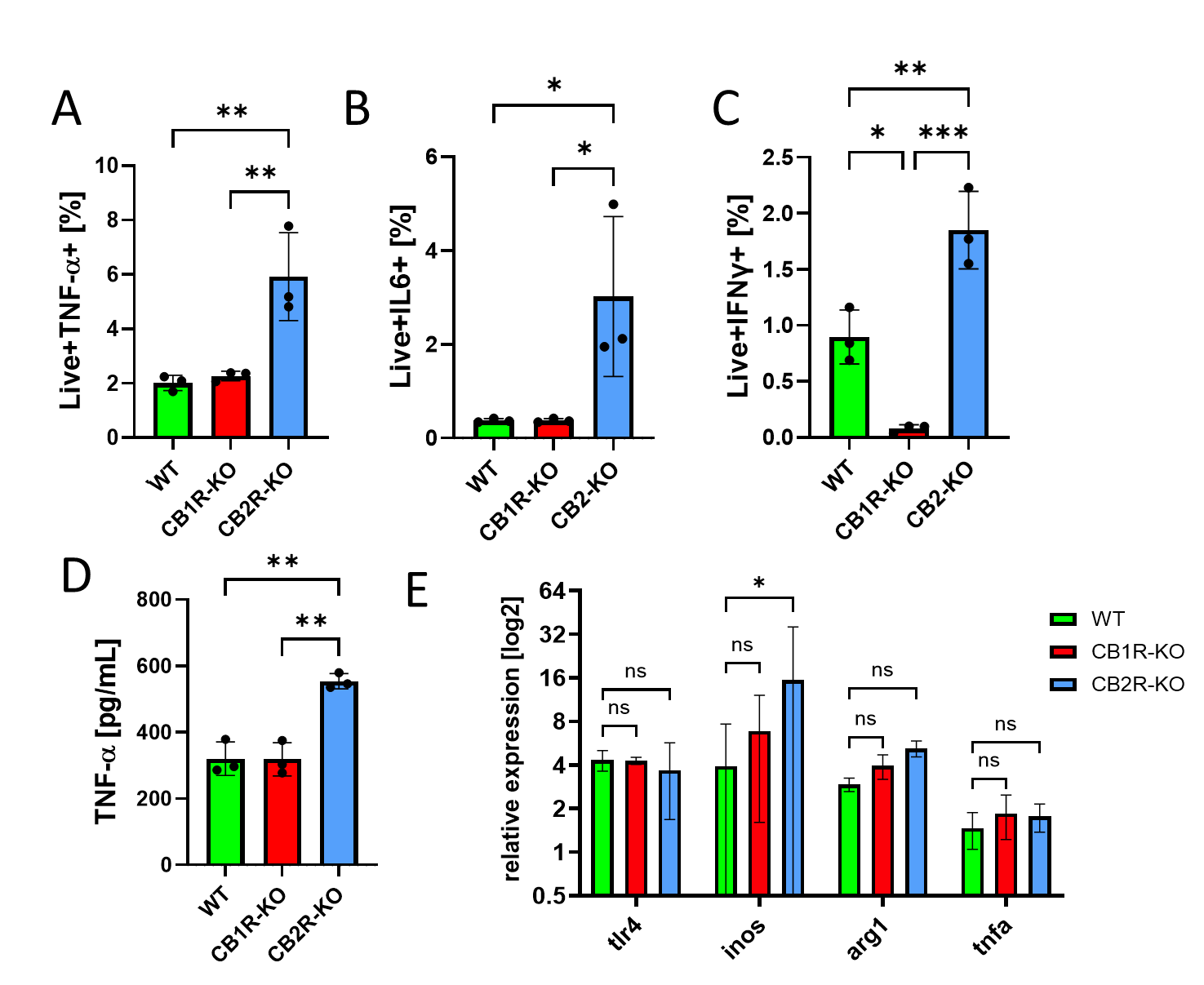


**Figure S5.** **Differential cytokine production by CB1R- and CB2R-deficient bone marrow–derived macrophages following *Salmonella* infection.** Bone marrow–derived macrophages (BMDMs) were isolated from wild-type (WT), CB1 receptor knockout (CB1R-KO), and CB2 receptor knockout (CB2R-KO) mice and infected in vitro with *Salmonella enterica* serovar Typhimurium (strain 12023) for 2 hours**. (A–C)** Flow cytometry analysis of intracellular TNF-α **(A),** IL-6 **(B),** and IFN-γ **(C)** expression in infected BMDMs across the three genotypes. **(D**) ELISA quantification of TNF-α levels in culture supernatants from infected BMDMs. **(E).** Quantitative PCR analysis of *Tlr4, Inos, Arg1, and Tnfa* mRNA expression in uninfected BMDMs from the indicated genotypes, assessing baseline transcriptional differences in innate immune mediators. Data represent mean ± SEM, with individual points indicating biological replicates from independent mice. Statistical comparisons were performed using one-way ANOVA followed by Tukey’s post hoc test. Significance: *p < 0.05, **p < 0.01, ***p < 0.001.


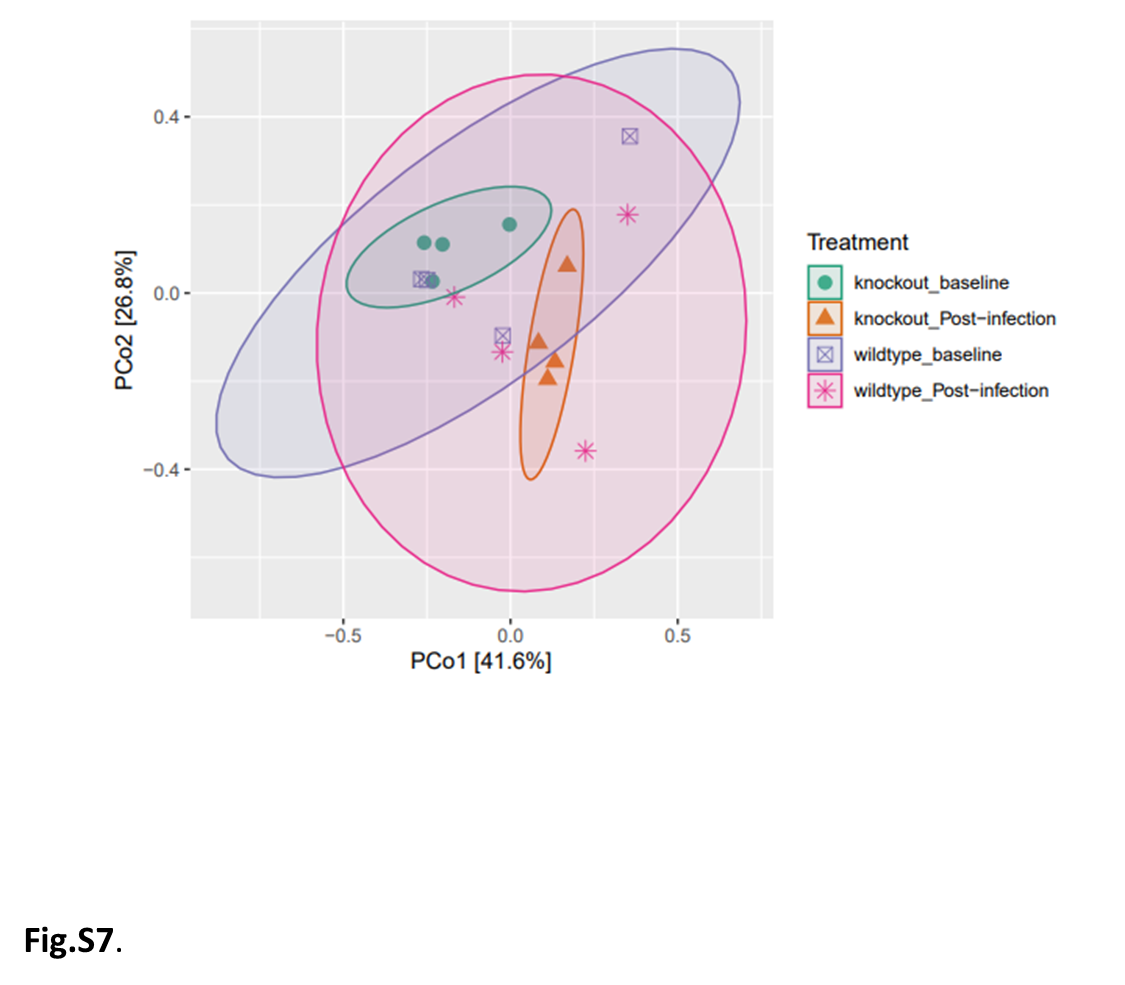


**Figure S6. Principal Coordinates Analysis (PCoA) of microbial community composition.** PCoA was performed using Bray–Curtis dissimilarity to assess beta diversity across four experimental groups: wild-type (WT), CB1 receptor knockout (CB1R-KO), and CB2 receptor knockout (CB2R-KO) mice and infected or not with *Salmonella* Typhimurium*.* Each point represents the microbial profile of one sample. Ellipses indicate 95% confidence intervals for each group. Axes represent the first two principal coordinates, explaining 41.6% and 26.8% of the total variance, respectively. PERMANOVA analysis showed significant differences in community composition between CB1R-KO baseline and CB1R-KO infected samples (R² = 0.53, p = 0.032), but no significant differences between other groups (p > 0.05).


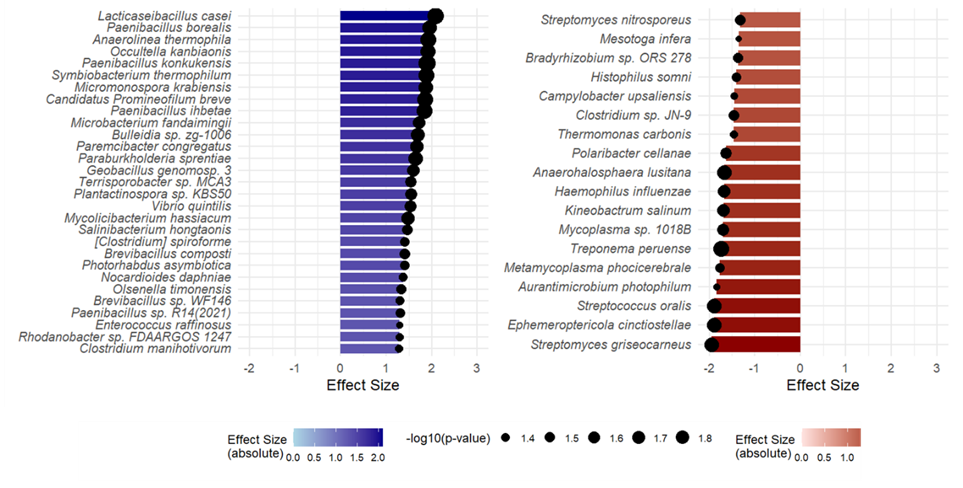


**Figure S7. Baseline gut microbiota composition differs between CB2R-KO and WT mice.** Differential abundance analysis of microbial taxa in CB2R knockout (CB2R-KO) and wild-type (WT) mice at baseline (pre-infection). Blue bars indicate taxa significantly enriched in CB2R-KO mice, while red bars indicate enrichment in WT mice. The length of each bar corresponds to the effect size, and the circle size represents -log10(p-value), reflecting the statistical significance of the difference. Microbial taxa shown met a significance threshold.

**Supplementary tables**

**Table S1. Differential gut microbiota in uninfected CB2R-KO mice compared to wild-type (WT) controls.** Differentially abundant microbial species between CB2R knockout (CB2R-KO) and WT mice under uninfected conditions, based on statistical analysis of relative abundance (showing only species with p-values < 0.05). Positive effect sizes indicate higher abundance in WT mice, while negative values indicate lower abundance in WT mice (i.e., enrichment in CB2R-KO mice). P-values were calculated using appropriate statistical methods for compositional microbiome data.

| **Effect** | **p-value** | **Species** |
| --- | --- | --- |
| 2.09461301 | 0.016419942 | *Lacticaseibacillus casei* |
| 1.9500251 | 0.023590036 | *Paenibacillus borealis* |
| 1.9279183 | 0.019063603 | *Anaerolinea thermophila* |
| 1.91716869 | 0.020523527 | *Occultella kanbiaonis* |
| 1.88795722 | 0.014631822 | *Paenibacillus konkukensis* |
| 1.88280659 | 0.019200332 | *Symbiobacterium thermophilum* |
| 1.86365563 | 0.023671723 | *Micromonospora krabiensis* |
| 1.84733133 | 0.01939435 | *Candidatus Promineofilum breve* |
| 1.84499514 | 0.018978666 | *Paenibacillus ihbetae* |
| 1.72230061 | 0.032593127 | *Microbacterium fandaimingii* |
| 1.70368267 | 0.026260831 | *Bulleidia sp. zg-1006* |
| 1.66588914 | 0.028250136 | *Paremcibacter congregatus* |
| 1.64872739 | 0.023929813 | *Paraburkholderia sprentiae* |
| 1.59599263 | 0.033633309 | *Geobacillus genomosp. 3* |
| 1.54589672 | 0.037769798 | *Terrisporobacter sp. MCA3* |
| 1.53952654 | 0.035104282 | *Plantactinospora sp. KBS50* |
| 1.52528588 | 0.03567719 | *Vibrio quintilis* |
| 1.47687768 | 0.030364217 | *Mycolicibacterium hassiacum* |
| 1.45716909 | 0.040074024 | *Salinibacterium hongtaonis* |
| 1.41077893 | 0.044905514 | *[Clostridium] spiroforme* |
| 1.40738619 | 0.040194609 | *Brevibacillus composti* |
| 1.40593884 | 0.043339715 | *Photorhabdus asymbiotica* |
| 1.37653862 | 0.046304981 | *Nocardioides daphniae* |
| 1.34053808 | 0.042288714 | *Olsenella timonensis* |
| 1.3024732 | 0.046600946 | *Brevibacillus sp. WF146* |
| 1.30215058 | 0.044381311 | *Paenibacillus sp. R14(2021)* |
| 1.29882132 | 0.048931478 | *Enterococcus raffinosus* |
| 1.29705911 | 0.047515001 | *Rhodanobacter sp. FDAARGOS 1247* |
| 1.27970949 | 0.047243162 | *Clostridium manihotivorum* |
| -1.3248141 | 0.038874446 | *Streptomyces nitrosporeus* |
| -1.3570921 | 0.049189491 | *Mesotoga infera* |
| -1.3631185 | 0.042929954 | *Bradyrhizobium sp. ORS 278* |
| -1.409225 | 0.045011988 | *Histophilus somni* |
| -1.4459969 | 0.048496804 | *Campylobacter upsaliensis* |
| -1.4642281 | 0.040955847 | *Clostridium sp. JN-9* |
| -1.4653563 | 0.047587962 | *Thermomonas carbonis* |
| -1.6275485 | 0.037155851 | *Polaribacter cellanae* |
| -1.6750265 | 0.023986638 | *Anaerohalosphaera lusitana* |
| -1.6751416 | 0.031530908 | *Haemophilus influenzae* |
| -1.6853897 | 0.031918984 | *Kineobactrum salinum* |
| -1.6954616 | 0.035341574 | *Mycoplasma sp. 1018B* |
| -1.7417026 | 0.020090427 | *Treponema peruense* |
| -1.7678427 | 0.044641191 | *Metamycoplasma phocicerebrale* |
| -1.8453395 | 0.048982214 | *Aurantimicrobium photophilum* |
| -1.8913793 | 0.025504946 | *Streptococcus oralis* |
| -1.8980907 | 0.024776446 | *Ephemeroptericola cinctiostellae* |
| -1.950164 | 0.023753328 | *Streptomyces griseocarneus* |

**Table S2. Differential gut microbiota in infected CB2R-KO mice compared to wild-type (WT) controls.** Differentially abundant microbial species between CB2R knockout (CB2R-KO) and WT mice under infected conditions, based on statistical analysis of relative abundance (showing only species with p-values < 0.05). Positive effect sizes indicate higher abundance in WT mice, while negative values indicate lower abundance in WT mice (i.e., enrichment in CB2R-KO mice). P-values were calculated using appropriate statistical methods for compositional microbiome data.

| **effect** | **p-value** | **species** |
| --- | --- | --- |
| 3.859164 | 0.003752 | *Limosilactobacillus reuteri* |
| 2.482954 | 0.012019 | *Lactobacillus acidophilus* |
| 2.433801 | 0.043101 | *Faecalitalea cylindroides* |
| 2.427716 | 0.009612 | *Thermophilibacter immobilis* |
| 2.342164 | 0.006799 | *Collinsella stercoris* |
| 2.222094 | 0.017555 | *Lactobacillus intestinalis* |
| 2.186714 | 0.01683 | *Bifidobacterium animalis* |
| 1.98508 | 0.036836 | *Leptogranulimonas caecicola* |
| 1.966884 | 0.011173 | *Levilactobacillus brevis* |
| 1.96591 | 0.017345 | *Olsenella sp. oral taxon 807* |
| 1.914523 | 0.019453 | *Streptomyces incarnatus* |
| 1.870418 | 0.0258 | *Sulfuriflexus mobilis* |
| 1.813473 | 0.031319 | *Mammaliicoccus stepanovicii* |
| 1.809254 | 0.021712 | *Streptomyces peucetius* |
| 1.793468 | 0.019977 | *Streptomyces phaeolivaceus* |
| 1.771369 | 0.027766 | *Desulfuromonas versatilis* |
| 1.759965 | 0.024658 | *Collinsella aerofaciens* |
| 1.750412 | 0.026085 | *Nocardia cyriacigeorgica* |
| 1.750301 | 0.023543 | *Comamonas thiooxydans* |
| 1.747648 | 0.03646 | *Paenibacillus sp. R14(2021)* |
| 1.701353 | 0.019995 | *Suicoccus acidiformans* |
| 1.67555 | 0.032139 | *Limosilactobacillus fermentum* |
| 1.671052 | 0.047338 | *Vibrio palustris* |
| 1.670792 | 0.024908 | *Paraburkholderia sprentiae* |
| 1.660302 | 0.028287 | *Achromobacter pestifer* |
| 1.64522 | 0.028993 | *Arthrobacter sp. NicSoilB4* |
| 1.635731 | 0.025347 | *Halobacillus halophilus* |
| 1.6218 | 0.029817 | *Streptococcus sp. Marseille-Q6470* |
| 1.621177 | 0.035643 | *Actinoplanes sp. N902-109* |
| 1.599131 | 0.037966 | *Haladaptatus salinisoli* |
| 1.597443 | 0.023203 | *Lactobacillus gasseri* |
| 1.579575 | 0.019945 | *Virgibacillus phasianinus* |
| 1.579332 | 0.025205 | *Parolsenella massiliensis* |
| 1.563837 | 0.029819 | *Mycobacterium senriense* |
| 1.559932 | 0.035843 | *Lactobacillus helveticus* |
| 1.556545 | 0.035437 | *Proteus sp. ZN5* |
| 1.536247 | 0.040078 | *Lactobacillus prophage Lj771* |
| 1.522036 | 0.044351 | *Streptococcus parauberis* |
| 1.5155 | 0.037113 | *Rhodococcus fascians* |
| 1.511237 | 0.027138 | *Amedibacterium intestinale* |
| 1.507483 | 0.036098 | *Melaminivora jejuensis* |
| 1.487471 | 0.046571 | *Pseudomonas sp. Seg1* |
| 1.48642 | 0.043436 | *Xanthomonas translucens* |
| 1.482214 | 0.042267 | *Lactobacillus iners* |
| 1.478858 | 0.041698 | *Symbiobacterium thermophilum* |
| 1.475707 | 0.02744 | *Lactobacillus paragasseri* |
| 1.434723 | 0.049845 | *Gordonia amarae* |
| 1.415214 | 0.041926 | *Parolsenella catena* |
| 1.411494 | 0.049944 | *Vagococcus fluvialis* |
| 1.410431 | 0.03465 | *Enterococcus wangshanyuanii* |
| 1.409487 | 0.043769 | *Cardiobacterium hominis* |
| 1.403889 | 0.047767 | *Bacillus rugosus* |
| 1.3987 | 0.040472 | *Erythrobacter neustonensis* |
| 1.385053 | 0.044034 | *Streptococcus canis* |
| 1.373219 | 0.037262 | *Methylotenera mobilis* |
| 1.365567 | 0.045745 | *Fictibacillus arsenicus* |
| 1.356855 | 0.042264 | *Bacillus sp. OxB-1* |
| 1.346039 | 0.03991 | *Lactobacillus crispatus* |
| 1.345834 | 0.049842 | *Glutamicibacter sp. JL.03c* |
| 1.329695 | 0.044913 | *Chania multitudinisentens* |
| 1.316348 | 0.045106 | *Mycetohabitans rhizoxinica* |
| 1.308129 | 0.047967 | *Tumebacillus avium* |
| 1.297817 | 0.045247 | *Amylolactobacillus amylophilus* |
| 1.284907 | 0.044601 | *Fusobacterium ulcerans* |
| 1.271263 | 0.042396 | *Xenorhabdus doucetiae* |
| 1.230383 | 0.041816 | *Paenibacillus protaetiae* |
| -1.30874 | 0.040528 | *Sphingobacterium spiritivorum* |
| -1.34059 | 0.044479 | *Adhaeribacter swui* |
| -1.3477 | 0.045952 | *Thiomicrorhabdus aquaedulcis* |
| -1.35098 | 0.049048 | *Leisingera sp. BMJM1* |
| -1.36347 | 0.043606 | *Flavobacterium sp. K5-23* |
| -1.3753 | 0.048457 | *Burkholderia sp. PAMC 26561* |
| -1.37621 | 0.043822 | *Methanospirillum hungatei* |
| -1.38065 | 0.049631 | *Salinibacter ruber* |
| -1.41991 | 0.039885 | *Mucilaginibacter ginsenosidivorax* |
| -1.43456 | 0.044726 | *Flavobacterium inviolabile* |
| -1.43561 | 0.048758 | *Arachidicoccus sp. B3-10* |
| -1.43801 | 0.045297 | *Hymenobacter sp. 5420S-77* |
| -1.4484 | 0.045731 | *Elizabethkingia meningoseptica* |
| -1.45291 | 0.0394 | *Chryseobacterium sp. 3008163* |
| -1.45434 | 0.045606 | *Flavobacterium crassostreae* |
| -1.45719 | 0.049326 | *Tatumella ptyseos* |
| -1.46334 | 0.035326 | *Ottowia testudinis* |
| -1.46371 | 0.042696 | *Deep-sea thermophilic phage D6E* |
| -1.46496 | 0.035678 | *Lacunisphaera limnophila* |
| -1.4674 | 0.041228 | *Kaistella flava (ex Peng et al. 2021)* |
| -1.4913 | 0.034719 | *Nocardioides rotundus* |
| -1.52652 | 0.038584 | *Nonlabens sp. Ci31* |
| -1.54228 | 0.042487 | *Paraburkholderia fungorum* |
| -1.55604 | 0.039254 | *Chryseobacterium sp. PCH239* |
| -1.56392 | 0.034406 | *Mucilaginibacter rubeus* |
| -1.57396 | 0.026701 | *Corynebacterium flavescens* |
| -1.58245 | 0.034161 | *Mucilaginibacter ginkgonis* |
| -1.59836 | 0.026153 | *Methylocaldum marinum* |
| -1.59932 | 0.03033 | *Flavobacterium kingsejongi* |
| -1.61474 | 0.040414 | *Colwellia sp. Arc7-635* |
| -1.62897 | 0.035 | *Micavibrio aeruginosavorus* |
| -1.64183 | 0.027637 | *Flavobacterium sangjuense* |
| -1.64638 | 0.029855 | *Nocardioides sp. TF02-7* |
| -1.64849 | 0.040786 | *Alistipes finegoldii* |
| -1.65471 | 0.029089 | *Pedobacter sp. SW-16* |
| -1.67248 | 0.036393 | *Qipengyuania aquimaris* |
| -1.69297 | 0.033417 | *Muricauda sp. SCSIO 64092* |
| -1.70141 | 0.029863 | *Capnocytophaga sp. oral taxon 864* |
| -1.70914 | 0.047653 | *Fulvivirga sp. W9P-11* |
| -1.71551 | 0.03361 | *Methylococcus sp. EFPC2* |
| -1.7211 | 0.03064 | *Arenibacter algicola* |
| -1.75791 | 0.021201 | *Chryseobacterium sp. 6424* |
| -1.82521 | 0.019388 | *Chryseobacterium lactis* |
| -1.92894 | 0.025678 | *Alistipes onderdonkii* |
| -1.95761 | 0.034083 | *Kaistella antarctica* |
| -1.99955 | 0.011186 | *Odoribacter splanchnicus* |
| -2.04097 | 0.020681 | *Chryseobacterium oryzae* |
| -2.05013 | 0.019308 | *Alistipes dispar* |
| -2.14015 | 0.012442 | *Opitutus sp. GAS368* |
| -2.36793 | 0.016644 | *Alistipes megaguti* |

**References:**

1. Schade R, Butler DSC, McKenna JA, Di Luccia B, Shokoohi V, Hamblin M, Pham THM, Monack DM. Transcriptional profiling links unique human macrophage phenotypes to the growth of intracellular *Salmonella enterica* serovar Typhi. Sci Rep. 2024;14(1):12811. Epub 20240604. doi: 10.1038/s41598-024-63588-6. PubMed PMID: 38834738; PMCID: PMC11150401.
